# In Vivo Glx Measurements From GABA‐Edited HERMES at 3 T Are Not Consistent With Those From Short‐TE PRESS Across Scanners, Brain Regions, Diagnostic and Age Groups

**DOI:** 10.1002/nbm.70171

**Published:** 2025-12-03

**Authors:** Alice R. Thomson, Viola Hollestein, Amy Goodwin, Anne Fritz, Beth Oakley, Declan Murphy, Edward Bullock, Ellen Demurie, Eva Loth, Giorgia Bussu, Herbert Roeyers, Isabel Yorke, Jan K. Buitelaar, Julia Koziel, Laura Colomar, Manon A. Krol, Matthew Bowdler, Nele Herregods, Pascal Aggensteiner, Pim Pullens, Rosemary Holt, Terje Falck‐Ytter, Tony Charman, Tomoki Arichi, Nicolaas A. Puts

**Affiliations:** ^1^ Department of Forensic and Neurodevelopmental Sciences, Institute of Psychiatry, Psychology, and Neuroscience King's College London London UK; ^2^ MRC Centre for Neurodevelopmental Disorders King's College London London UK; ^3^ Autism Research Centre University of Cambridge Cambridge UK; ^4^ Research in Developmental Diversity Lab, Department of Experimental‐Clinical and Health Psychology Ghent University Belgium; ^5^ Development and Neurodiversity Lab, Department of Psychology Uppsala University Uppsala Sweden; ^6^ Department of Child and Adolescent Psychiatry King's College London London UK; ^7^ Department of Cognitive Neuroscience, Donders Institute for Brain, Cognition and Behaviour Radboud University Nijmegen Medical Center Nijmegen Netherlands; ^8^ Department of Radiology and Nuclear Medicine Ghent University Hospital Ghent Belgium; ^9^ Central Institute of Mental Health Department of Child and Adolescent Psychiatry and Psychotherapy Mannheim Germany; ^10^ Department of Women's and Children's Health, Center of Neurodevelopmental Disorders (KIND), Centre for Psychiatry Research Karolinska Institutet & Stockholm Health Care Services, Region Stockholm Stockholm Sweden; ^11^ Centre for the Developing Brain King's College London London UK

**Keywords:** 1H‐MRS, ACC, agreement, edited 1H‐MRS, Glx, HERMES, PRESS, Thalamus

## Abstract

1H‐Magnetic resonance spectroscopy (1H‐MRS) is a noninvasive technique for quantifying brain metabolites, including glutamate, glutathione (GSH), and γ‐aminobutyric acid (GABA), which are essential for brain function and implicated in various neurodevelopmental conditions. As such, 1H‐MRS methods that enable reliable and accurate measurement of these metabolites are of considerable clinical value. Hadamard Encoding and Reconstruction of MEGA‐Edited Spectroscopy (HERMES; echo time [TE] = 80 ms) is a spectral editing technique that allows for the simultaneous quantification of GABA and GSH, using subtraction approaches to resolve these metabolites in a difference spectrum. Additionally, glutamate plus glutamine resonances (Glx) can be resolved either from the HERMES GABA‐edited difference spectrum (GABA‐DIFF) or from the sum of all HERMES transients (SUM spectrum). However, the reliability of 80‐ms HERMES for quantification of Glx has not been systematically assessed. Here, we evaluate the agreement between Glx obtained from HERMES GABA‐DIFF and SUM spectra with Glx derived from short‐TE PRESS (TE = 35 ms), which is conventionally used for Glx estimation and has demonstrated reproducibility. Data were acquired from 139 participants across two brain regions (ACC and Thalamus voxels), three scanners, two diagnostic groups (autism and neurotypical development) and two age groups (adolescent/adult and preschooler). Comparisons were made using both creatine‐scaled and tissue‐corrected Glx estimates. Our findings demonstrate significant systematic and proportional bias between Glx estimates from HERMES (SUM and GABA‐DIFF) and short‐TE PRESS, consistent across scanners, voxels, age groups and diagnostic categories. These findings indicate that Glx estimates derived from HERMES are not directly comparable to those from short‐TE PRESS, and this discrepancy is consistent across a multisite study setting. This underscores the importance of sequence selection and careful methodological consideration when integrating and interpreting data from 1H‐MRS across different acquisition protocols.

## Graphical abstract

We evaluate the agreement between Glx (Glutamine plus Glutamine) estimates obtained from HERMES GABA‐DIFF (blue) and SUM spectra with Glx derived from short‐TE PRESS (TE = 35ms; green). Data was acquired from 139 participants, across two voxels (ACC and thalamus), three different scanners and multiple age and diagnostic groups (adults/preschoolers and individuals with/without autism). Overall, HERMES (DIFF & SUM) and PRESS Glx estimates show poor correlation and agreement, a finding consistent across scanners, voxels, diagnostic and age groups.

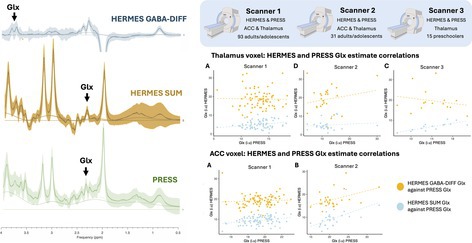

**Abbreviations**
1H‐MRS: 1H‐magnetic resonance spectroscopy
ACC: anterior cingulate cortex
CoV: coefficients of variation
CSF: cerebral spinal fluid
FWHM: full‐width‐at‐half‐maximum
GABA: γ‐aminobutyric acid
GABA‐DIFF: HERMES GABA‐edited difference spectrum
Glx: the combined signal of glutamate and glutamine
GM: grey matter
GSH: glutathione
HERMES: Hadamard Encoding and Reconstruction of MEGA‐Edited Spectroscopy
i.u: institutional unit
ICC: intraclass correlation coefficients
IQR: interquartile range
LEAP: Longitudinal European Autism Project
LoA: limits of agreement
MEGA‐PRESS: MEshcher‐GArwood Point RESolved Spectroscopy sequence
ms: milliseconds
NAA: N‐acetylaspartate
OFF_GABA_
: non‐GABA editing pulse
OFF_GSH_
: non‐GSH editing pulse
ON_GABA_
: GABA‐editing pulse
ON_GSH_
: GSH‐editing pulse
PIP: Preschool Brain Imaging and Behaviour Project
PRESS: Point Resolved Spectroscopy
QM: quality metrics
Rho: Spearman's rank correlation coefficient
semi‐LASER: semi‐Localization through Adiabatic Selective Refocusing
SNR: signal‐to‐noise ratio
SUM: sum of all HERMES transients
T: Tesla
T1: longitudinal relaxation
T2: transverse relaxation
tCr: total creatine
TD: typically developing
TE: echo time
TI: inversion time
TR: repetition time
WM: white matter

## 1 Introduction

1H‐Magnetic resonance spectroscopy (1H‐MRS) is a powerful noninvasive tool for investigating the neurochemical composition and activity of the human brain in vivo [1, 2, 3, 4, 5, 6]. Among other metabolites, 1H‐MRS allows for the quantification of glutamate and γ‐aminobutyric acid (GABA), the adult mammalian brain's major excitatory and inhibitory neurotransmitters, respectively. Glutamate and GABA play essential roles in neurotransmission and neuronal metabolism [7, 8], and as such are relevant for typical brain development, cognitive function, learning, behaviour and aging [2, 6, 9, 10, 11, 12, 13, 14]. The ability to quantify glutamate and/or GABA is of further importance as alterations in their levels have been identified in several neurological and neurodevelopmental conditions including autism [3, 15, 16, 17], epilepsy [18, 19, 20] and schizophrenia [21, 22, 23]. 1H‐MRS approaches that facilitate accurate, accessible and efficient measurement of these brain metabolites in clinical cohorts are therefore highly valuable for the continued characterisation of atypical neurodevelopment and function, as well as how therapeutic approaches modulate brain chemistry [24, 25].

Glutamate is conventionally measured with single voxel, short echo‐time (TE) approaches such as Point Resolved Spectroscopy (PRESS [26]), Stimulated Echo Acquisition Mode (STEAM [27]) and semi‐Localization through Adiabatic Selective Refocusing (semi‐LASER [28]; TE approximately 25–40 ms). Short TE approaches achieve poor chemical shift dispersion at standard 1.5 to 3 Tesla (T) scanner field strengths, meaning that glutamate resonances at 3.75 ppm, 2.3 ppm and 2.1 ppm overlap with that of its precursor, glutamine, and so cannot be accurately resolved [4]. As a result, the combined concentration of glutamate and glutamine, denoted ‘Glx’, is commonly reported across studies. However, the ratio of glutamate to glutamine contributing to Glx appears to vary with scanning parameters and spectral fitting approaches utilised [29, 30, 31]. Moreover, the contribution of glutamate and glutamine to Glx might change due to age‐related or pathological changes in glutamate neuronal and extraneuronal compartmentalisation [7, 14]; however, this is poorly characterised.

Existing approaches for the individual quantification of glutamate and glutamine include TE‐averaged PRESS, which uses variable TEs across a single acquisition to generate a TE‐averaged spectrum in which glutamate signals are better separated from glutamine [32]. Another approach, two‐dimensional (2D) J‐resolved spectroscopy (JPRESS [30, 33]) encodes for a second J‐frequency dimension to reduce spectral overlap and thus allows for more accurate fitting of glutamate. Additionally, long‐TE PRESS (TE = 80 ms) can be used for glutamate only quantification by minimising overlapping glutamine signals due to differences in J‐modulation effects at longer TEs [34]. However, compared to conventional sequences (specifically short‐TE PRESS), glutamate/glutamine only acquisition approaches demonstrate reduced repeatability [35, 36], as well as requiring longer scan times and tailored processing pipelines (with more complex basis sets and corrections) and, importantly, are not widely available for all scanner vendors. Moreover, other clinically and functionally interesting metabolites cannot be quantified by these methods, including creatine, choline and N‐acetylaspartate (NAA) [34, 36]. This hinders their usage.

Resonances of lower‐concentration metabolites including GABA (1–3 mM) and glutathione (GSH; 1–2 mM) are masked by metabolites in higher concentrations (e.g., NAA; 15–20 mM or creatine; 10–15 mM [37, 38, 39, 40]). To address this, J‐difference ‘editing’ techniques such as MEshcher‐GArwood Point RESolved Spectroscopy (MEGA‐PRESS [41]) are commonly used for their measurement. MEGA‐PRESS uses alternating ‘edit‐ON’ and ‘edit‐OFF’ acquisitions, which exploit the coupling of molecular resonances, which for GABA, are at 3.02 ppm, 2.3 ppm and 1.9 ppm (at a GABA‐specific optimal TE of 68 ms or at a TE of 80 ms suboptimally for MM‐suppression or when using multiplexed editing [2]). During the ‘edit‐ON’ acquisition, a 1.9‐ppm editing pulse selectively modulates the 1.9 ppm GABA resonance and, due to J‐coupling, the GABA resonance at 3.02 ppm. The overlapping creatine signal is unaffected by the editing pulse. During the ‘edit‐OFF’ acquisition, an off‐resonance editing pulse (typically at 7.5 ppm) has no effect within the range of resonant frequencies of interest. Subtraction of the edited spectra (edit‐ON) from unedited spectra (edit‐OFF) gives the difference (DIFF) spectra, which contain only metabolite resonances modulated by the GABA‐selective editing, including GABA at 3.02 ppm [4]. Because of contamination with macromolecules (at 1.7 ppm), which are also affected by the GABA‐selective editing pulse, the GABA signal is often denoted as GABA+ (GABA + macromolecules [4]). Glutamate/Glx measurements can also be obtained from MEGA‐PRESS acquisitions, as the bandwidth of the 1.9‐ppm editing pulse is broad enough to partially refocus the coupling partners of glutamate and glutamine resonances at 2.1 ppm. As a result, Glx resonances co‐edit with GABA and can be quantified from the GABA‐DIFF spectrum (at 3.75 and 2.34 ppm). Glx can also be measured at 7.46 ppm in the MEGA‐PRESS edit‐OFF spectrum [30, 31, 42].

Hadamard Encoding and Reconstruction of MEGA‐Edited Spectroscopy (HERMES [43]) is an extension of MEGA‐PRESS that facilitates the dual editing of metabolites (e.g., GABA and GSH) within a single acquisition. Similar to MEGA‐PRESS, HERMES uses interleaved ‘editing’ and ‘nonediting’ pulses that constitute 4 subexperiments: a GABA‐editing (1.9 ppm) and GSH‐editing (4.56 ppm) cosine–sine–Gaussian editing pulse (ON_GABA_, ON_GSH_; *experiment A)*, a GABA‐editing pulse (ON_GABA_, OFF_GSH_; *experiment B*), a GSH‐editing pulse (OFF_GABA_, ON_GSH_; *experiment C*) and a nonediting pulse (OFF_GABA_, OFF_GSH_; *experiment D*). Akin to MEGA‐PRESS, glutamine and glutamate resonances are co‐edited during the GABA editing acquisitions and can be quantified at 3.75 ppm in the GABA difference (GABA‐DIFF) spectrum (A + B—C—D). Alternatively, Glx can be resolved from the sum of all transients (A + B + C + D; SUM), which resembles the PRESS spectrum, although at a longer TE of 80 ms. Because of the smaller number of averages per subspectra (usually 60 averages standard), the SUM spectra may be preferable for Glx quantification for maximum signal‐to‐noise ratio (SNR) compared to the GABA‐DIFF spectra. However, the utility of 80‐ms HERMES Glx (from GABA‐DIFF or SUM) compared to conventional Glx acquisition approaches remains undefined. Thus, at present, for the measurement of Glx and GABA in a single participant, it is recommended that both HERMES and PRESS are acquired [42, 44]. In practice, this leads to longer scan times, increasing the risk of motion artefacts and early termination. This is particularly challenging for populations with poorer scan tolerance, such as individuals with neurodevelopmental conditions [45].

Furthermore, prior work investigating Glx quantification from GABA‐edited sequences (namely, MEGA‐PRESS) has focused on single scanner analyses, with small, homogeneous, predominantly adult samples [30, 31, 42, 43, 46]. It is well established that GABA and Glx change with development [47, 48] and across numerous clinical conditions [49], which may impact the consistency of Glx quantification. Understanding Glx quantification and identifying condition‐specific, age‐specific, and scanner‐specific differences is thus of strong relevance for transparency in reporting.

Here, we aimed to evaluate the agreement between Glx obtained from HERMES GABA‐DIFF and HERMES SUM spectra with Glx derived from short‐TE PRESS, which is conventionally used for Glx estimation and has demonstrated accuracy and reproducibility (TE = 35 ms [34, 35, 50, 51, 52]). Short‐TE PRESS has been previously used as a ‘standard’ approach for assessing the validity of MEGA‐PRESS Glx measurements [42]. We evaluated systematic differences between raw Glx estimates (linear scaling in a defined direction); however, the agreement between raw values was less relevant to our analysis since our primary focus was on consistency between measures (e.g., are Glx measurements correlated even if there is a systematic difference between them). Therefore, we also examined proportional biases between estimates, which indicate that the difference between estimates changes with Glx magnitude. Unlike systematic differences, proportional biases are less easily corrected for and so pose a significant challenge when comparing metabolite data across different acquisition methods. Data were acquired from 139 participants across two voxels (anterior cingulate cortex [ACC] and thalamus), three scanners, two diagnostic groups (autism and typical development), and two age groups (adolescent/adult and preschool child) to assess agreement across biological, technical and demographic variability, conditions reflective of realistic clinical, multisite study designs.

This evaluation is relevant for reducing scan times in paediatric and clinical population and in multimodal studies where more than just 1H‐MRS is acquired, whereby a single HERMES acquisition could serve to simultaneously measure multiple edited metabolites (GABA, GSH) and Glx, instead of multiple acquisitions (e.g., HERMES and PRESS).

## 2 Methods

### 2.1 Participants

Data were acquired as part of the Longitudinal European Autism Project (LEAP) and Preschool Brain Imaging and Behaviour Project (PIP), part of the AIMS‐2‐TRIALS [53, 54] clinical research programme (https://www.aims‐2‐trials.eu/). Our sample consisted of 139 participants, 70 diagnosed with autism and 69 typically developing (TD). Participants had an age range of 3–36 years. Data were acquired from 3 scanners at 2 sites; Radboud University Nijmegen Medical Centre (RUNMC, Netherlands; henceforth referred to as Scanner 1 and Scanner 3; note that these were two different scanners, see Supplementary Table S1) and the Central Institute of Mental Health (Mannheim, Germany; henceforth referred to as Scanner 2). For scanner features per site please refer to Supplementary Table S1. Demographics are presented in Table 1 for the data included (after exclusion of poor‐quality data; see below). Scanners 1–2 acquired data from adolescent and adult participants (age 13–36 years; Table 1), while scanner 3 acquired data from preschooler age participants only (age 2–4 years; Table 1). Data were collected with a research protocol that was approved by the local medical‐ethics committees [54] and after written informed consent from the volunteers or a legal guardian.

**TABLE 1:** Participant demographics.

Scanner	Participants	Age (median (IQR): range)	Sex (M(F))	Diagnosis (autism (TD))
Scanner 1	93	21 (8): 14–36 years	65 (28)	53 (40)
Scanner 2	31	21 (7): 13–31 years	23 (8)	15 (16)
Scanner 3	15	4 (0.15): 3.4–4.6 years	6 (9)	2 (13)
Total	139	21 (9.08): 3.4–36 years	94 (45)	70 (69)

Abbreviations: F = Female, IQR = interquartile range, M = male.

### 2.2 MRS Data Acquisition

**FIGURE 1:**
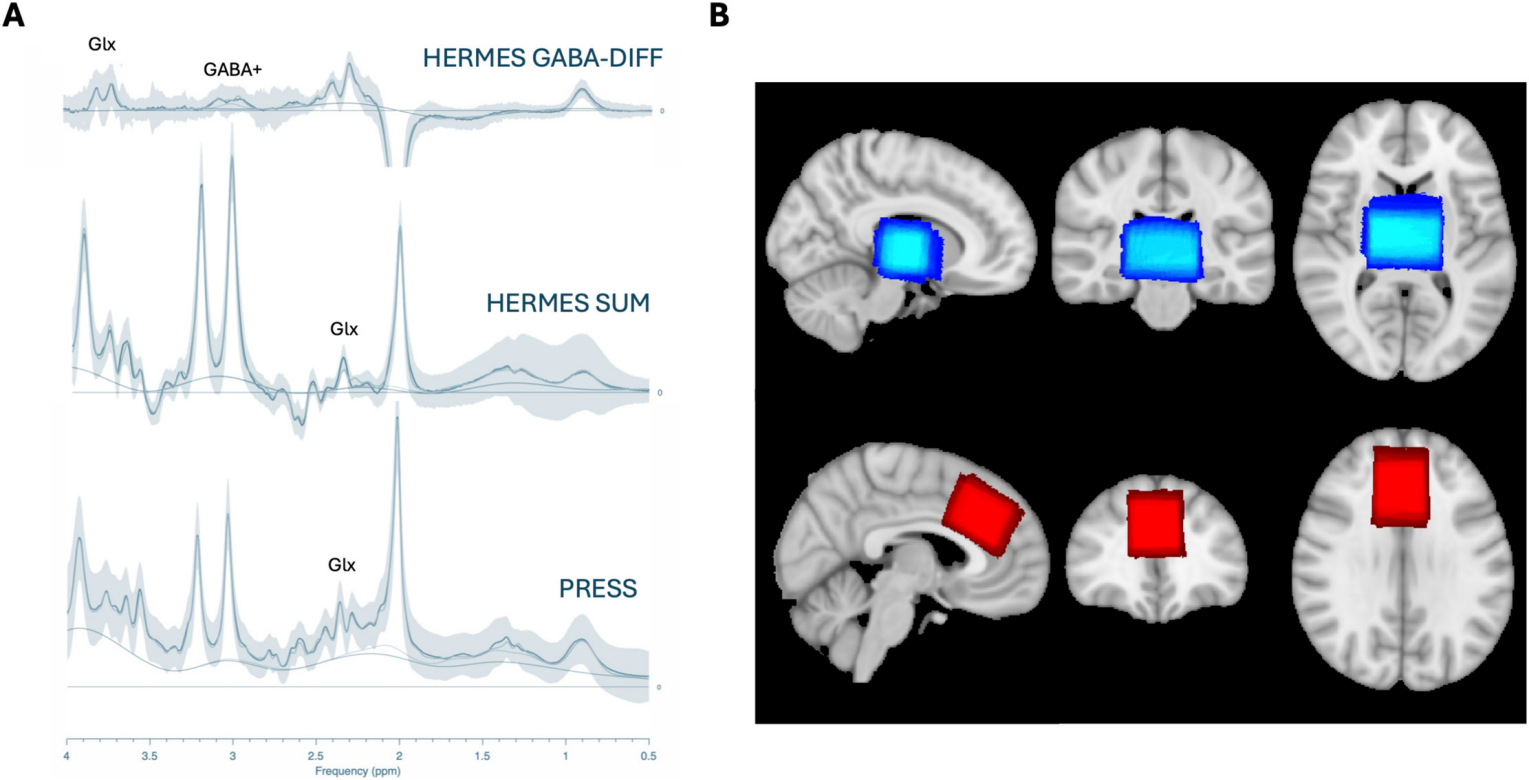
**Voxel placement and mean HERMES and PRESS spectra.** (**A**) Mean HERMES GABA‐DIFF, HERMES SUM and PRESS spectra showing model fit, model baseline and median residual fits (error bars). (**B**) Standard T1‐weighed image displaying heat plots of the MRS voxels on the thalamus (blue) and ACC (red), created by normalising T1‐weighted scans and then voxels to a standard space (MNI52 T1 1 mm brain) and calculating the overlap. Heat plots are shown on a standard anatomical image (MNI52 T1 1 mm brain). Greater intensity (brighter) indicates increased overlap and thus consistency in MRS voxel placement between participants.

Data were collected at two sites on 3 3 T MRI scanners. For a summary of scanner details and sequence acquisition parameters per site, see Supplementary Table S1. First, a T1‐weighted MP‐RAGE anatomical image was acquired for voxel placement and tissue segmentation (TR/TE/TI = 2300/3/900 ms, voxel size = 1.1 × 1.1 × 1.2 mm, flip angle = 9–11°, matrix size = 256 × 256, FOV = 270 mm, 176–208 slices). MRS data were acquired from a 26 mm × 40 mm × 24 mm (scanner 1 and scanner 3) or 30 mm × 30 mm × 30 mm (scanner 2) thalamus voxel and a 35 mm × 30 mm × 25 mm anterior cingulate cortex (ACC) voxel (scanner 1 and 2). Both voxels were centred on the midline. The thalamus voxel was rotated such that the superior edge was aligned with the inferior edge of the lateral ventricles (coronal slice), avoiding the ventricle and corpus callosum. The ACC voxel was rotated such that the inferior‐anterior vertex was vertically aligned with the most frontal part of the corpus callosum (sagittal plane), while the inferior edge was parallel with the corpus callosum (see Figure 1). MRS data were first acquired using vendor native PRESS (TR/TE: 2000/35 ms, 64 averages, 4096 data points sampled at 4000‐Hz spectral bandwidth), followed by Universal HERMES; 20‐ms editing pulses placed at 1.90 ppm, 4.56 ppm and 7.46 ppm in the ON_GABA_, ON_GSH_ and OFF conditions, respectively, TR/TE = 2000/80 ms, 240 averages (60 averages per subexperiment), 4096 data points sampled at 4000 Hz spectral bandwidth [43]. Both short TE and HERMES TE water unsuppressed acquisitions were acquired (16 transients), as per recommendations [5]. Scanners 1–3 were Siemens. Note no ACC voxel was acquired at scanner 3 due to greater limitations on scan times (preschoolers). Summary HERMES GABA‐DIFF, HERMES SUM and PRESS spectra are shown in Figure 1, and mean spectra per scanner are shown in Supplementary Figure S1.

### 2.3 Data Processing

Raw MRS data (HERMES and PRESS spectra) were processed using Osprey (Version 2.4.0 [55]), an automated software for MRS analysis based in Matlab (version 2022a). Using Osprey, the following preprocessing steps were applied: coil combination, probabilistic spectral alignment for frequency and phase correction of individual transients, weighted averaging, Fourier transformation, eddy current correction using the water reference scan [56], and HSVD residual water signal removal [57]. For HERMES, subspectra were aligned using residual water peaks or the 2.01 ppm NAA peak before subspectra were subtracted or combined to calculate the GABA DIFF (A + B−C−D) and SUM (A + B + C + D) spectra. Averaged PRESS and HERMES spectra were modelled with TE‐specific simulated basis sets and a flexible spline baseline based on MR scanner vendor and scan sequence parameters (generated in the MATLAB toolbox FID‐A [55, 58]). Basis sets for macromolecule and lipid contributions were integrated as Gaussian basis functions [55]. All spectra were modelled between 0.5 and 4 ppm with linear baseline correction and a knot spacing of 0.55 ppm according to the Osprey model algorithm [55]. Modelling was performed for 19 metabolites (ascorbic acid, aspartic acid, total creatine, creatine methylene, GABA+, glycerophosphocholine, glutathione, glutamine, glutamate, myo‐inositol, lactate, total N‐acetylaspartate, n‐acetylaspartylglutamate, total choline, phosphocholine, phosphocreatine, phosphatidylethanolamine, scyllo‐inositol, taurine), five macromolecules and three lipids (MM09, MM12, MM14, MM17, MM20, Lip09, Lip13, Lip20) for all spectra. Glx was quantified from the HERMES DIFF, HERMES SUM and PRESS spectra.

The Osprey co‐registration module (via SPM version 12) was used to register the MRS voxel to the T1‐weighted images acquired at the scan. Segmented T1‐weighted images were used to obtain *tissue‐corrected* water‐scaled (molar) estimates of metabolite concentrations in institutional unit (i.u), whereby water‐reference‐ratio metabolite concentrations are scaled according to the assumption that metabolite concentrations in cerebral spinal fluid (CSF) are negligible [2, 59]. Further corrections for tissue‐specific water concentrations (grey matter [GM], white matter [WM] and CSF) and tissue‐specific water and metabolite longitudinal and transverse relaxation were performed [59], as outlined in consensus papers [44, 60]. As well as estimated tissue‐corrected concentrations, Glx levels were estimated relative to total creatine (tCr) levels measured within the same voxel (e.g., Glx/tCr; *creatine‐scaled*). For edited‐MRS approaches, creatine was estimated from the edit‐OFF spectrum. T1‐weighted images and voxel masks were also registered to a standard MN1152 T1 1‐mm brain anatomical image for the creation of voxel heat plots (Figure 1).

### 2.4 Spectral Artefacts and Quality Control

Spectra were visually assessed by experienced MRS data users (AT, NP). MRS spectra with significant artifacts due to motion and/or lipid contamination and/or spurious echoes were excluded. As per MRSinMRS [44], quantitative quality metrics (QM) were also assessed including signal‐to‐noise ratio (SNR) of the creatine peak, linewidth of the creatine peak expressed in Hertz (Hz; full‐width‐at‐half‐maximum; FWHM) and frequency shift for alignment (Hz). Fit residuals were also computed separately for each spectrum (HERMES GABA‐DIFF, HERMES SUM and PRESS), based on the deviation between the average spectra and the optimised model. The Osprey model algorithm utilised for modelling the HERMES and PRESS spectra currently does not provide individual metabolite QM (Osprey Version 2.4.0 [55]). Reported QM are thus based on average model fits of the HERMES (GABA‐DIFF & HERMES SUM) and PRESS spectra.

Per scanner, quantitative QM (SNR, FWHM, frequency shift and fit residuals) that deviated three times the interquartile range above the third quartile or below the first quartile were identified, and the corresponding spectra inspected again to confirm whether the data were of poor quality before exclusion. Overall, a total of 33 datasets were excluded (19% of data; 25 thalamus voxels, 8 ACC voxels), comprising: 28 datasets from scanner 1 (14% of data; 20 thalamus, 8 ACC), 4 datasets from scanner 2 (6% of data; 4 thalamus) and 1 dataset from scanner 3 (6% of data; 1 thalamus). Note that data exclusion rates were not higher for scanner 3, which is the preschooler child cohort, likely because scans were acquired during natural sleep. Adult/adolescent scans were awake. Detailed reasons (and n numbers) for data exclusion are shown in Supplementary Table S2.

### 2.5 Statistical Analysis

**FIGURE 2:**
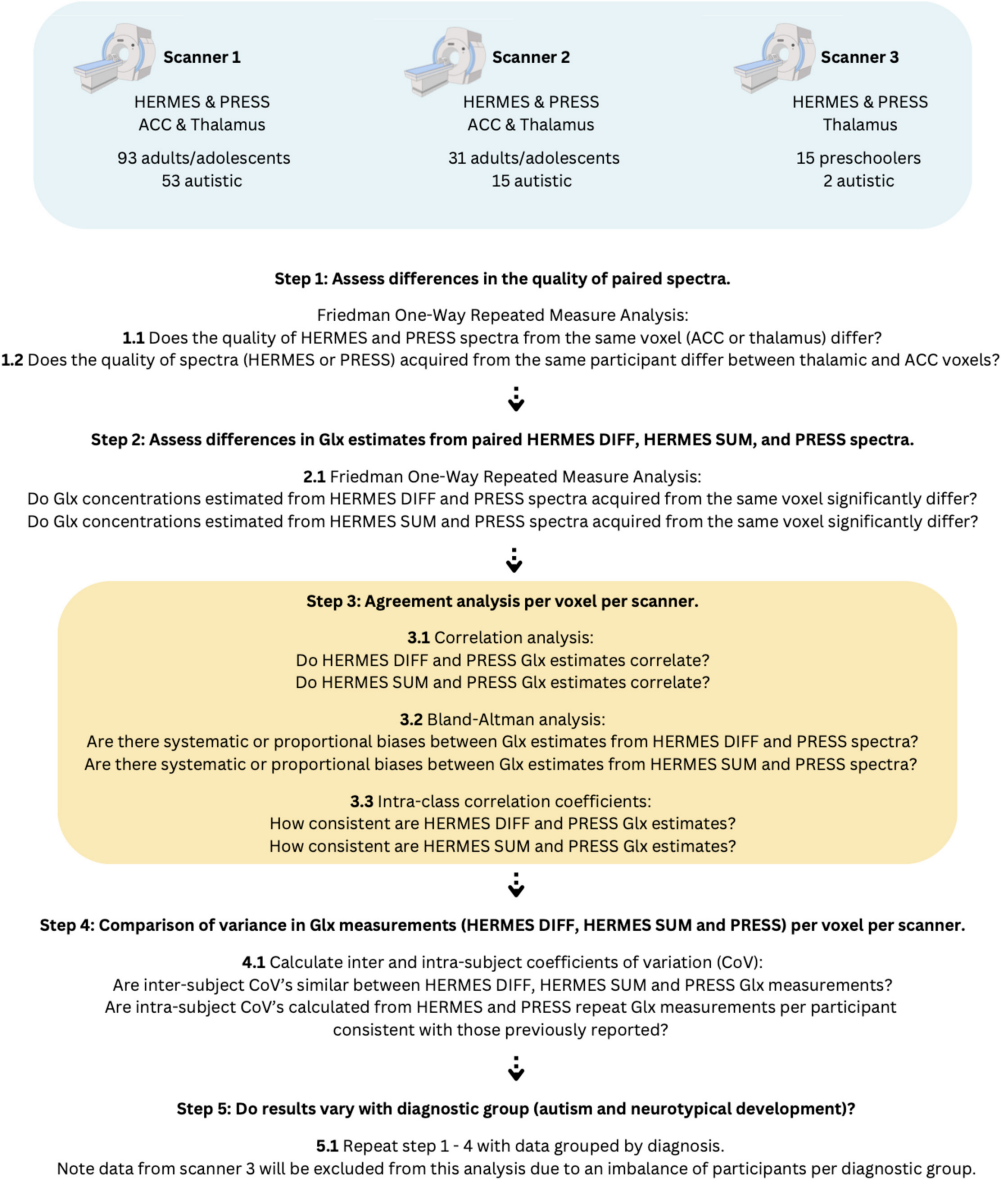
**Summary of** **the acquisition and analysis workflow used**. Statistical analysis methods and results are labelled accordingly.

Statistical analysis of data was performed on RStudio. Quantitative MRS QM and Glx data were not normally distributed for all scanners (as assessed by Shapiro–Wilk test and Q–Q plots). As such, all statistical procedures described are suitable for nonparametric data. Note that, in all cases where appropriate, repeat‐measure statistical tests were performed on paired measures (HERMES and PRESS Glx/QM from the same voxel and participant) as opposed to testing group means. Thus, trends identified are at the participant level. Statistical analysis is presented below according to the workflow shown in Figure 2.

#### 2.5.1 Step 1: Assess Differences in the Quality of Paired HERMES and PRESS Spectra

Friedman one‐way repeated measure analyses were used to assess if QM significantly differed between paired HERMES GABA‐DIFF, HERMES SUM and PRESS spectra acquired from the same voxel and participant (*step 1.1*) and between paired voxels (HERMES or PRESS spectra acquired from thalamus and ACC voxels in the same participant; *step 1.2*). Analysis was performed per scanner (scanner 1–3). Pairwise Wilcoxon rank sum tests were used for post hoc testing, with Bonferroni corrections. Statistical significance was assessed at a Bonferroni‐adjusted threshold of *p*_adjusted_ < 0.05, with exact adjusted *p* values reported.

Associations between Glx estimates and spectral QM were assessed using Spearman's rank correlation coefficients (rho). We chose to examine only the relationship between Glx and FWHM, as QMs are colinear.

#### 2.5.2 Step 2: Assess Differences in Glx Estimates From Paired HERMES GABA‐DIFF, HERMES SUM and PRESS Spectra

Friedman one‐way repeated measure analyses were used to assess if Glx estimates significantly differed between paired HERMES GABA‐DIFF, HERMES SUM and PRESS spectra acquired from the same voxel and participant, and between paired voxels (HERMES or PRESS Glx estimated from thalamus and ACC voxels in the same participant). Analysis was performed per scanner. Pairwise Wilcoxon rank sum tests were used for post hoc testing, with Bonferroni correction for multiple comparisons. Statistical significance was assessed at a Bonferroni‐adjusted threshold of *p*_adjusted_ < 0.05, with exact adjusted *p* values reported.

#### 2.5.3 Step 3: Agreement Analysis

Spearman's rank partial correlation coefficients (rho) calculated using the *ppcor* package in R were used to observe the correlation between HERMES spectra (GABA‐DIFF & SUM) and PRESS spectra Glx estimates per voxel and per scanner, while controlling for FWHM (*step 3.1*). Statistical significance was assessed at a Bonferroni‐adjusted threshold of *p*_adjusted_ < 0.05, with exact adjusted *p* values reported. Correlations were compared between scanners 1–3 using Fisher's *r*‐to‐*z* transform and *z*‐test on the partial correlation coefficients. Significant *p* values (*p* < 0.05) indicate correlations are significantly different between scanners.

Bland–Altman plots are a graphical analysis tool used to assess the agreement of two paired methods/measurements (*step 3.2*). A scatter plot shows the difference between paired Glx measurements (method 1 − method 2) on the y‐axis, plotted against the mean of the paired Glx measurements (method 1 + method 2 / 2) on the x‐axis. For example, (HERMES GABA‐DIFF Glx − PRESS Glx) plotted against (HERMES GABA‐DIFF Glx + PRESS Glx / 2). Horizontal lines on the plot mark the mean difference of the two measures (estimated bias) and the limits of agreement (LoA), which are calculated as the mean difference ± 1.96 times the standard deviation of the mean difference. If differences are normally distributed, 95% of the data points should lie within this range if the methods show ‘agreement’ [61]. Also calculated are the 95% confidence intervals for the mean difference and LoA, which describe the precision of these estimates [61]. If 0 (line of equality) is not within the 95% confidence interval for the mean difference, then there is a significant systematic bias between Glx measurements between the two methods.

In this study, Giavarina adaptations of Bland–Altman were used [62], which are identical to the aforementioned method but with the exception that the percentage difference (with respect to the mean) is plotted instead of the raw difference between paired Glx measures. For example, ((HERMES GABA‐DIFF Glx − PRESS Glx/PRESS Glx) × 100) plotted against (HERMES GABA‐DIFF Glx + PRESS Glx / 2). This accounts for the possible presence of heteroscedastic variance, e.g., the variability of paired measurement differences changes with the magnitude of the dependent variable (Glx concentration). Prior to Giavarina plots, differences between paired measurements were tested for normality using the Wilks–Shapiro test. Giavarina plots were assessed per scanner per voxel (*step 3.2*).

To test for proportional bias between Glx measurements, linear regression analyses (model = percentage difference of paired measures ~ mean of paired measures) were performed. A significant beta coefficient would indicate a proportional bias; as Glx concentration changes, the magnitude of the difference between paired measurements changes (*step 3.2*).

Intraclass correlation coefficients (ICCs) were also used to assess the agreement of Glx measures (*step 3.3*), reflecting both the correlation between measures and the degree of agreement between measures using a group analysis (e.g., do subjects keep their rank order between repeat measures). ICCs are calculated as a value between 0 and 1. In this study, we used a fixed two‐way ICC formula for single measurement (model 3 [63, 64]), whereby the consistency between paired Glx measurements is assessed, with agreement between raw values being less relevant. ICC values (with 95% confidence intervals) were calculated per scanner per voxel. An ICC value of 0–0.50 represents poor consistency between measures, an ICC of 0.50–0.75 represents moderate consistency between measures, an ICC of 0.75–0.90 represents good consistency, and an ICC greater than 0.90 indicates excellent consistency between two measures [63].

#### 2.5.4 Step 4: Comparison of Variance in Glx Measurements per Voxel per Scanner

Coefficients of variation (CoVs) were calculated and used to assess the variance of Glx measurements. Intersubject CoVs, calculated as the group standard deviation ($\sigma_{\text{group}}$) divided by group mean ($\mu_{\text{group}}$) times by 100 [65], facilitate the comparison of Glx measurement variability irrespective of differences in the magnitude between the different methods (HERMES GABA‐DIFF, HERMES PRESS and SUM). Intersubject CoVs were calculated for HERMES and PRESS Glx measurements per scanner and per voxel. As the true sample variance should be similar for equivalent methods (measuring the same dependent variable from the same population), higher intersubject CoVs for a Glx measure (e.g., HERMES GABA‐DIFF Glx/HERMES SUM Glx/PRESS Glx) may indicate increased nuisance variance in that acquisition approach [30].

Intrasubject CoVs were also calculated as a measure of the variance in repeat Glx measures per participant (HERMES GABA‐DIFF, HERMES SUM and PRESS), using the standard deviation ($\sigma_{\text{subject}}$) and mean ($\mu_{\text{subject}}$) of the three Glx measurements per subject (HERMES SUM, HERMES GABA‐DIFF and PRESS [65]). Mean intrasubject CoV's were computed for the thalamus and ACC voxels for each scanner. This facilitated assessment of whether, on average, the three approaches (HERMES SUM, HERMES GABA‐DIFF and PRESS) produced Glx estimates within the ‘normal’ range of repeatability for MRS measurements in the same participant (per voxel per scanner).

$$\text{Intersubject}\;\mathrm{CoV}=100\frac{\sigma_{\text{group}}}{\mu_{\text{group}}}$$

$$\text{Intrasubject}\;\mathrm{CoV}=\frac{1}{n}\sum_{s=1}^{n}100\frac{\sigma_{\text{subject}}}{\mu_{\text{subject}}}$$

#### 2.5.5 Step 5: Do Results Vary With Diagnostic Group (Autism and Typical Development)

The analyses described in steps 1–4 were repeated with data grouped by diagnosis (TD or autism). Note data from scanner 3 (TD = 13, autism = 2) were excluded from these analyses due an imbalance of participants per diagnostic group.

## 3 Results

### 3.1 Step 1. Assess Differences in the Quality of Paired Spectra per Scanner

QMs of thalamus and ACC PRESS and HERMES spectra per scanner are reported in Supplementary Table S3. Note that significant differences in QM of HERMES and PRESS spectra from scanner 1 and scanner 3 were observed, these being at the same site (but different scanners). Significant correlations between FWHM and HERMES SUM Glx estimates were also observed per scanner (Supplementary Table S4). As such, we control for FWHM where possible in all following analyses, which we consider sufficient to account for quality‐related effects as QMs are highly colinear (SNR, FWHM, fit resiudals etc).

#### 3.1.1 Quality of Paired PRESS and HERMES (GABA‐DIFF & SUM) Data Acquired From the Same Voxel Varies

Differences in quantitative QM of paired PRESS and HERMES (GABA‐DIFF & SUM) spectra acquired from the same voxel (ACC or thalamus) in the same participant were assessed (*step 1.1*). Results are displayed in Supplementary Figures S2 & S3 (with exact *p*_adjusted_ values reported). Overall, the fit residuals of the HERMES SUM spectra were significantly greater than those of paired PRESS spectra from the same ACC or thalamic voxel, whereas the fit residuals of the HERMES GABA‐DIFF spectra were significantly smaller than those of paired PRESS spectra from the same ACC or thalamic voxel. Differences in other QM of paired PRESS and HERMES spectra aquired from the same voxel (SNR, FWHM and frequency shift) varied across scanners; please refer to Supplementary Figures S2 & S3.

#### 3.1.2 The Quality of HERMES (GABA‐DIFF & SUM) and PRESS Data Acquired From the Same Participant Differs Between Thalamic and ACC Voxels

Friedman one‐way repeated measure analyses were used to assess if the quality of MRS data acquired from the same participant significantly differed between ‘paired’ voxels (thalamus and ACC; Supplementary Figure S4; *step 1.2*). In general, the SNR of HERMES and PRESS data acquired from the ACC was significantly greater than that of the HERMES and PRESS data from the thalamus per participant, while the FWHM of HERMES and PRESS data acquired from the ACC was significantly smaller than that of the thalamus per participant. Generally, fit residuals of HERMES GABA‐DIFF and SUM spectra were significantly greater in data from the ACC compared to the thalamus, although differences varied per scanner (Supplementary Figure S4).

### 3.2 Step 2. Assess HERMES GABA‐DIFF, HERMES SUM and PRESS Glx Estimates per Voxel per Scanner

Median creatine‐scaled and tissue‐corrected Glx concentrations per scanner (1–3) are shown in Table 2 (see Supplementary Figure S5 for specific significant differences in Glx estimates between scanners).

**TABLE 2:** Thalamus and ACC Glx Quantified From PRESS, HERMES GABA‐DIFF and HERMES SUM per Scanner.

	Glx/tCr – PRESS	Glx/tCr – HERMES GABA‐DIFF	Glx/tCr – HERMES SUM	Glx (i.u) – PRESS	Glx (i.u) – HERMES GABA‐DIFF	Glx (i.u) – HERMES SUM
Thalamus						
Scanner 1	1.14 (0.22)	1.77 (0.55)	0.47 (0.31)	13.38 (3.01)	18.21 (5.61)	4.79 (3.03)
Scanner 2	0.92 (0.18)	2.18 (1.13)	0.50 (0.26)	11.37 (2.55)	19.36 (7.40)	4.63 (1.97)
Scanner 3	0.96 (0.26)	1.68 (0.42)	0.65 (0.31)	14.11 (4.47)	18.37 (3.29)	7.30 (2.40)
*Kruskal–Wallis test for differences between scanners*	*H* (3) *= 62.49, p*_adjusted_ *= 0.00*	*H* (3) *= 63.51, p*_adjusted_ *= 0.00*	*H* (3) *= 9.075, p*_adjusted_ *= 0.06*	*H* (3) *= 76.69, p*_adjusted_ *= 0.00*	*H* (3) *= 67.33, p*_adjusted_ *= 0.00*	*H* (3) *= 54.72, p*_adjusted_ *= 0.00*
ACC						
Scanner 1	1.39 (0.14)	1.83 (0.24)	0.93 (0.24)	19.29 (2.22)	18.85 (3.10)	9.29 (2.65)
Scanner 2	1.42 (0.10)	1.71 (0.13)	0.90 (0.37)	20.09 (2.42)	16.79 (1.93)	8.99 (3.83)
*Kruskal–Wallis test for differences between scanners*	*H* (2) *= 11.318, p*_adjusted_ *= 0.006*	*H* (2) *= 30.16, p*_adjusted_ *= 0.00*	*H* (2) *= 2.8506, p*_adjusted_ *= 0.48*	*H* (2) *= 31.51, p*_adjusted_ *= 0.00*	*H* (2) *= 28.54, p*_adjusted_ *= 0.00*	*H* (2) *= 16.917, p*_adjusted_ *= 0.00*

Median (IQR) HERMES GABA‐DIFF, HERMES SUM and PRESS Glx concentrations (tissue‐corrected [i.u.] and creatine‐scaled). Differences in Glx concentrations between scanners were assessed using Kruskal–Wallis tests; the calculated *H* statistics and corresponding Bonferroni adjusted *p* values are displayed in the table, with *p*_adjusted_ < 0.05 indicating a significant difference in Glx concentrations across scanners. Note scanner 3 did not record data from an ACC voxel.

#### 3.2.1 Glx Estimates Acquired From the Same Voxel Differ Significantly Across Acquisition Approaches

**FIGURE 3:**
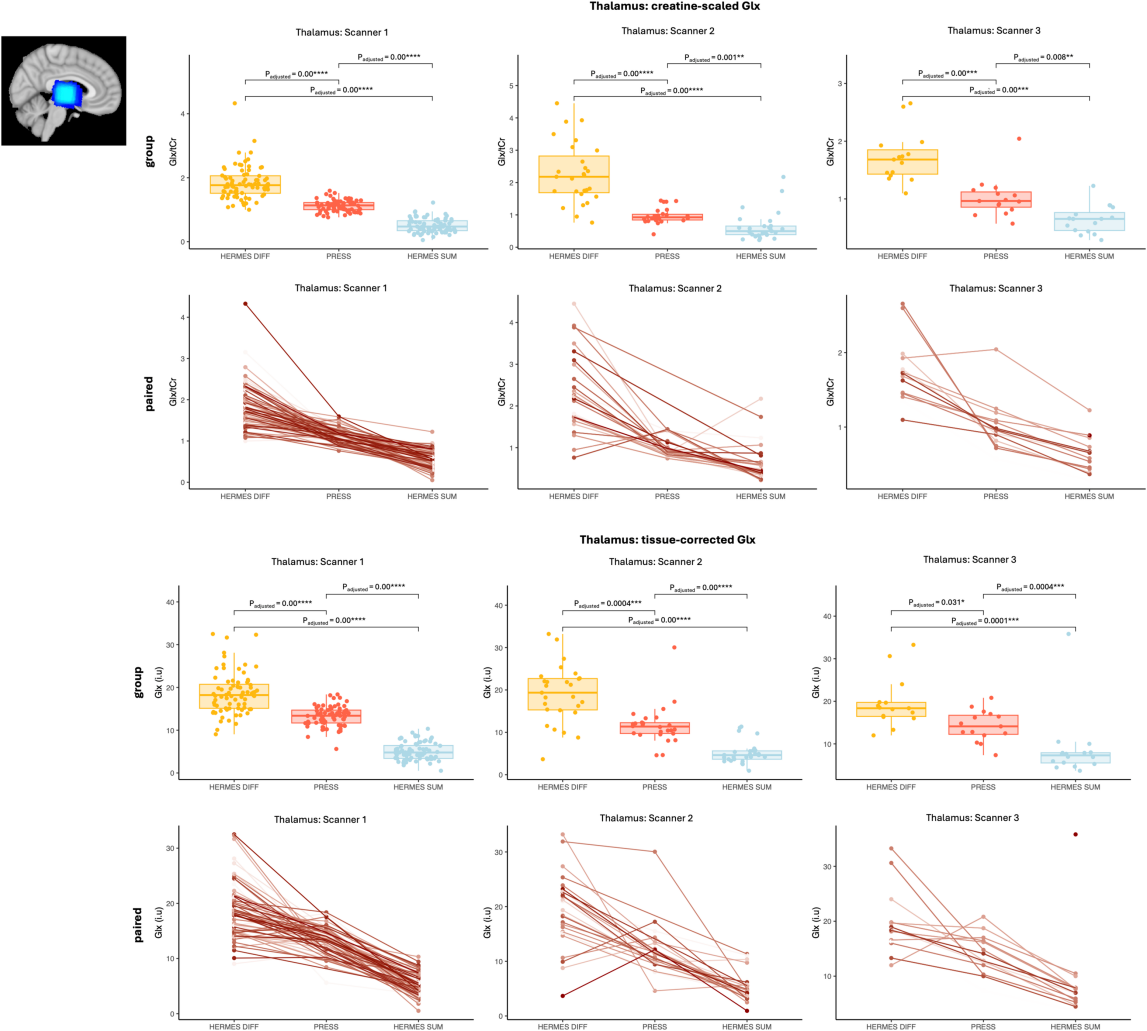
**Thalamus Glx concentrations estimated from paired HERMES GABA‐DIFF, HERMES SUM and PRESS spectra.** Tissue corrected (i.u) and creatine‐scaled (/tCr) thalamus Glx concentrations estimated from the HERMES GABA‐DIFF (DIFF), HERMES SUM (SUM) and PRESS spectra are shown per scanner. Significant differences in paired Glx estimates across acquisition approaches after multiple comparison correction are indicated per scanner; *p_adjusted_ < 0.05, **p_adjusted_ < 0.01, ***p_adjusted_ < 0.001, ****p_adjusted_ < 0.0001. Also shown are spaghetti plots, which show paired thalamic Glx measurements from the same participant (HERMES GABA‐DIFF, HERMES SUM and PRESS).

**FIGURE 4:**
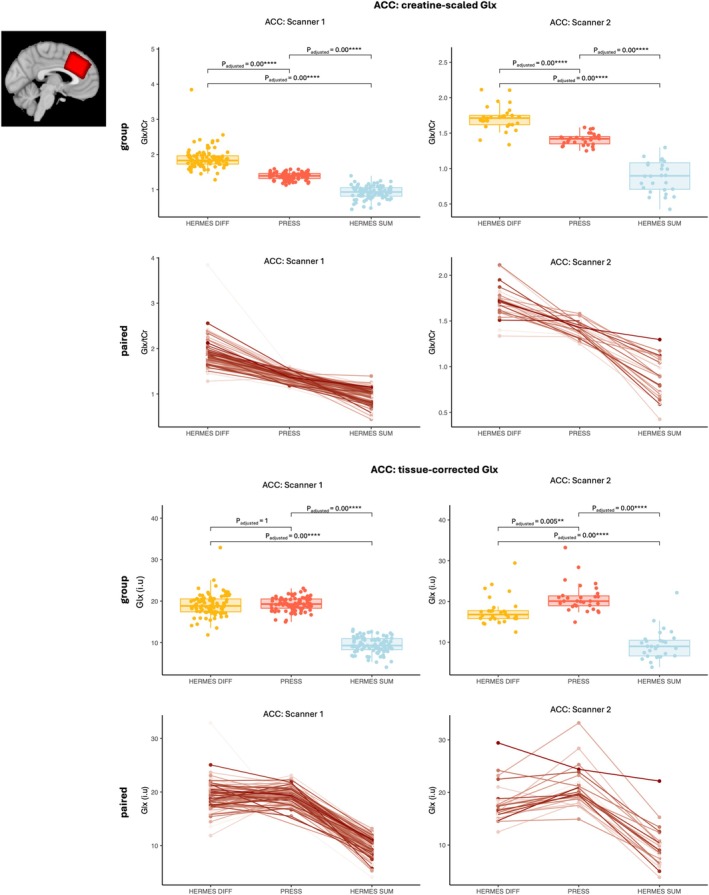
**ACC Glx estimated from paired HERMES GABA‐DIFF, HERMES SUM and PRESS spectra.** Tissue corrected (i.u) and creatine‐scaled (/tCr) ACC Glx concentrations estimated from the HERMES GABA‐DIFF (DIFF), HERMES SUM (SUM) and PRESS spectra are shown per scanner. Significant differences in paired Glx estimates across acquisition approaches after multiple comparison correction are indicated per scanner; *p_adjusted_ < 0.05, **p_adjusted_ < 0.01, ***p_adjusted_ < 0.001, ****p_adjusted_ < 0.0001. Also shown are spaghetti plots, which show paired ACC Glx measurements from the same participant (HERMES GABA‐DIFF, HERMES SUM and PRESS).

Differences between intrasubject paired Glx estimates from HERMES (GABA‐DIFF & SUM) and PRESS spectra acquired from the same voxel were assessed (*step 2.1*). Results are shown in Figures 3 and 4. In general, creatine‐scaled and tissue‐corrected HERMES GABA‐DIFF Glx estimates were significantly greater than paired PRESS Glx estimates, while creatine‐scaled and tissue‐corrected HERMES SUM Glx estimates were significantly smaller than paired PRESS Glx estimates from thalamus and ACC voxels. Note that there was one exception in that ACC tissue‐corrected GABA‐DIFF Glx estimates were significantly smaller than tissue‐corrected PRESS Glx estimates from scanner 3 (Figure 4).

### 3.3 Step 3. Agreement Analysis

#### 3.3.1 HERMES (GABA‐DIFF & SUM) and PRESS Glx Estimates Poorly Correlate

**FIGURE 5:**
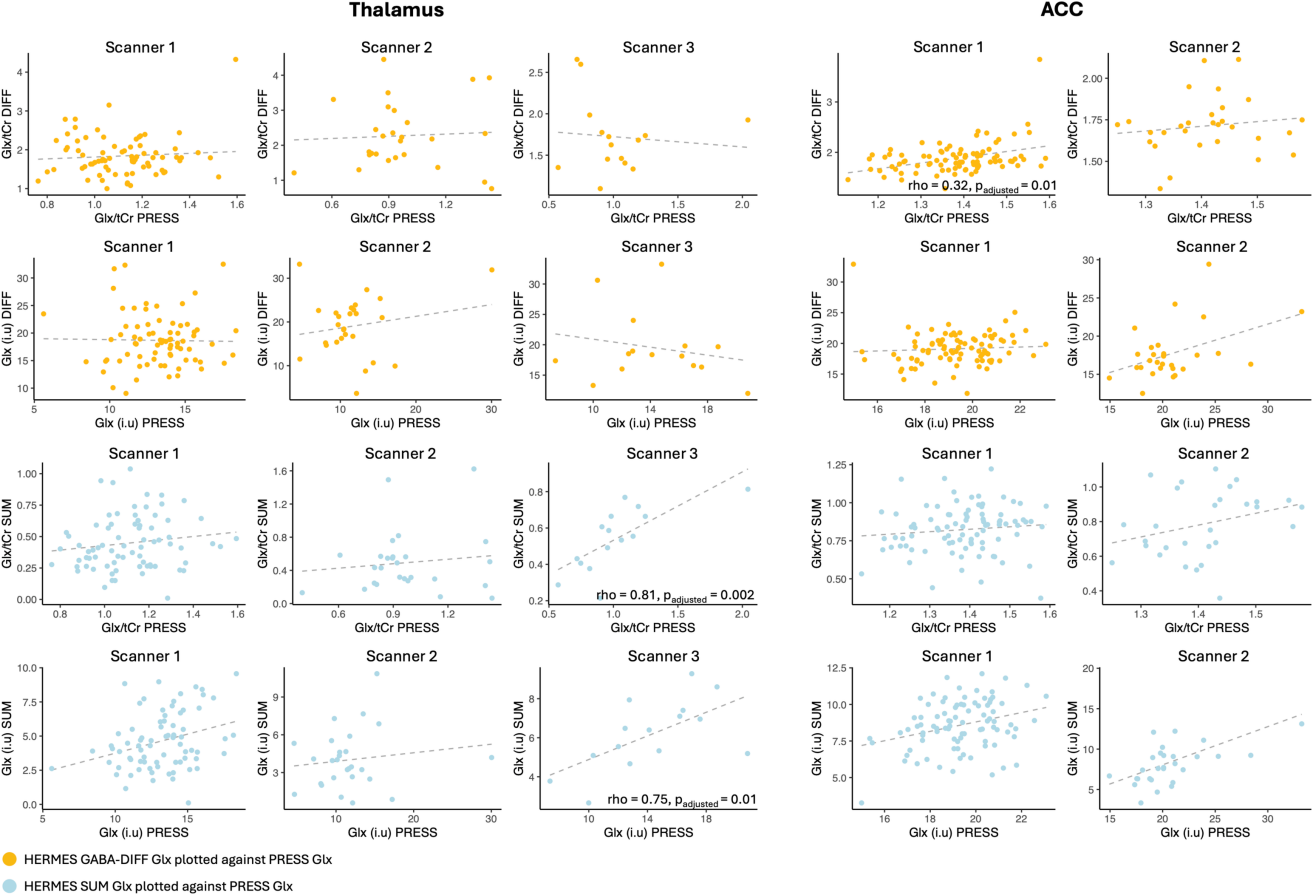
**Correlations between HERMES (SUM & GABA‐DIFF) and PRESS Glx estimates from the thalamus and ACC voxels.** Spearman's rank partial correlation coefficients (rho) were calculated between Glx estimated from HERMES (GABA‐DIFF & SUM) and PRESS from the thalamus and ACC voxels while controlling for FWHM, per scanner and for tissue‐corrected (i.u.) and creatine‐scaled (/tCr) data. Where significant after Bonferroni correction, partial correlation coefficients are displayed on the graph. Yellow points = correlation between HERMES GABA‐DIFF and PRESS Glx estimates, light blue points = correlation between HERMES SUM and PRESS Glx estimates. Grey line = linear association.

Partial Spearman rank correlation coefficients (rho) were calculated to assess the correlation between thalamus and ACC HERMES (GABA‐DIFF and SUM) and PRESS Glx estimates while controlling for FWHM (*step 3.1*). Results are described per voxel below and are plotted in Figure 5.

##### 3.3.1.1 Thalamus

No significant correlation was observed between thalamic Glx estimates from HERMES SUM and PRESS from scanner 1 or 2. In contrast, a significant partial correlation was observed in data from scanner 3 (preschoolers) between thalamic HERMES SUM and PRESS Glx estimates (ρ_creatine‐scaled_ = 0.81, *p*_adjusted_ = 0.002; ρ_tissue‐corrected_ = 0.75, *p*_adjusted_ = 0.01). Using Fisher's *r*‐to‐*z* transformation and *z*‐tests on the partial correlation coefficients, the correlation between HERMES SUM and PRESS Glx in scanner 3 data significantly differed from the correlation between HERMES SUM and PRESS Glx in scanner 2 data (tissue‐corrected: *z* = −2.89, *p*_adjusted_ = 0.011; creatine‐scaled: *z* = −3.45, *p*_adjusted_ = 0.00), and the correlation between HERMES SUM and PRESS Glx in scanner 1 data (tissue‐corrected: *z* = −3.268, *p*_adjusted_ = 0.002; creatine‐scaled: *z* = −3.87, *p*_adjusted_ = 0.00). No significant correlation was observed between thalamic Glx estimates from HERMES GABA‐DIFF and PRESS for any scanner.

##### 3.3.1.2 ACC

No significant correlation was observed between ACC Glx estimates from HERMES SUM and PRESS in data from scanner 1 or scanner 2; however, a significant and positive correlation was observed between HERMES GABA‐DIFF and PRESS Glx estimates in creatine‐scaled data from scanner 1 (ρ_creatine‐scaled_ = 0.32, *p*_adjusted_ = 0.01). No significant correlation was observed between Glx estimates from HERMES GABA‐DIFF and PRESS spectra from scanner 2.

#### 3.3.2 Bland–Altman Analyses Indicate Poor Agreement Between Paired HERMES (SUM & GABA‐DIFF) and PRESS Glx Estimates

##### 3.3.2.1 HERMES GABA‐DIFF Versus PRESS Glx estimates

**FIGURE 6:**
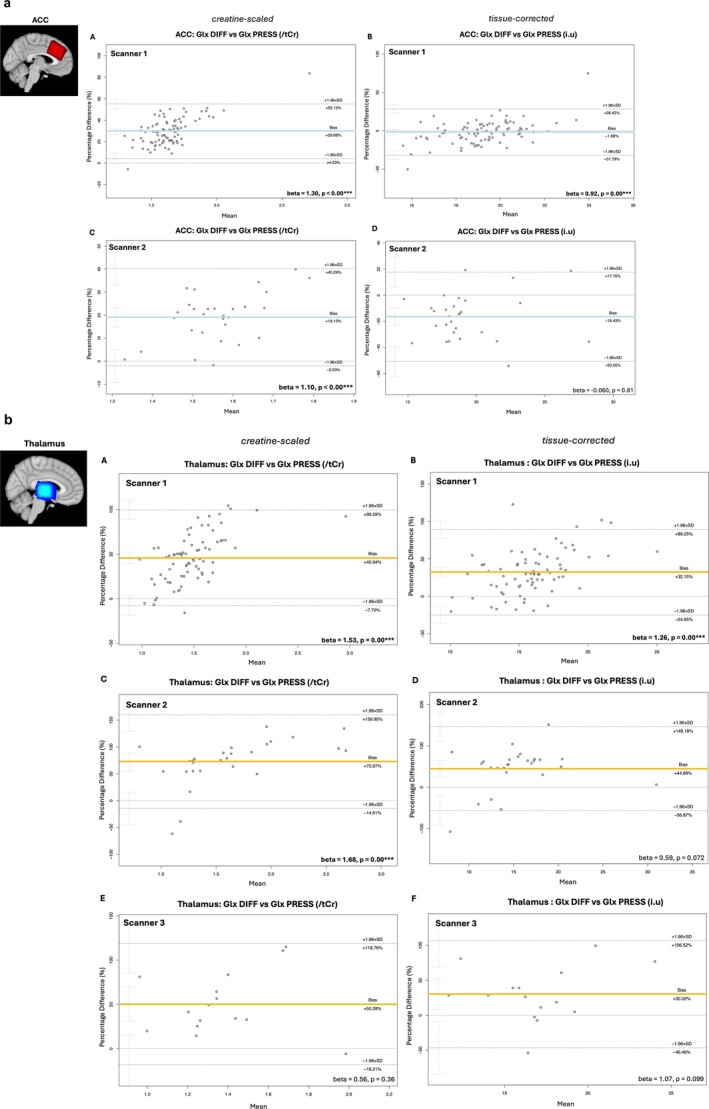
**(A) Bland–Altman (Giavarina version) plots comparing ACC Glx concentrations estimated from paired HERMES GABA‐DIFF and PRESS spectra per scanner.** For each plot, the percentage difference between paired measures is shown on the y‐axis, while the mean of the paired measures is shown the x‐axis. Solid blue lines represent the overall mean percentage difference (estimated bias) while dashed black lines represent the upper and lower limits of agreement (overall mean difference ± 1.96 standard deviation). Confidence intervals (95%) for limits of agreement are also shown. Linear regressions (model = percentage difference of paired measured ~ mean of paired measures) were used to identify proportional bias between paired measures, the resulting beta‐coefficient and corresponding *p* value is shown in the right‐hand corner of each plot; *p < 0.05, **p < 0.01, ***p < 0.001, ****p < 0.0001. **(B) Bland–Altman (Giavarina version) plots comparing thalamus Glx concentrations estimated from paired HERMES GABA‐DIFF and PRESS spectra per scanner.** For each plot, the percentage difference between paired measures is shown on the y‐axis, while the mean of the paired measures is shown the x‐axis. Solid yellow lines represent the overall mean percentage difference (estimated bias) while dashed black lines represent the upper and lower limits of agreement (overall mean difference ± 1.96 standard deviation). Confidence intervals (95%) for limits of agreement are also shown. Linear regressions (model = percentage difference of paired measured ~ mean of paired measures) were used to identify proportional bias between paired measures, the resulting beta‐coefficient and corresponding *p* value is shown in the right‐hand corner of each plot*;* *p < 0.05, **p < 0.01, ***p < 0.001, ****p < 0.0001.

Bland–Altman plots (Figure 6A,B; *step 3.2*) show that HERMES GABA‐DIFF Glx estimates are significantly systematically greater than paired PRESS Glx estimates. This was observed for creatine‐scaled and tissue‐corrected data and in both thalamus and ACC voxels. Note an exception: for scanner 3, ACC HERMES GABA‐DIFF tissue‐corrected Glx estimates were smaller than paired PRESS Glx estimates. Systematic bias between paired HERMES GABA‐DIFF and PRESS Glx estimates was proportionally greater in creatine‐scaled data compared to tissue‐corrected data (Figure 6A,B).

Regression analysis found significant and positive *proportional* bias between paired PRESS and HERMES GABA‐DIFF Glx estimates from the thalamus in data from scanner 1 (β_creatine‐scaled_ = 1.53, *p* = 0.00; β_tissue‐corrected_ = β = 1.26, *p* = 0.00) and scanner 2 (β = 1.68, *p* = 0.00; true for creatine‐scaled estimates only). This was also true for the ACC voxel from scanner 1 (β_creatine‐scaled_ = 1.30, *p* = 0.00; β_tissue‐corrected_ = 0.92, *p* = 0.00) and scanner 2 (β_creatine‐scaled_ = 1.10, *p* = 0.00). No significant proportional bias was observed between paired thalamic HERMES GABA‐DIFF and PRESS Glx estimates from scanner 3.

##### 3.3.2.2 HERMES SUM Versus PRESS Glx Estimates

**FIGURE 7:**
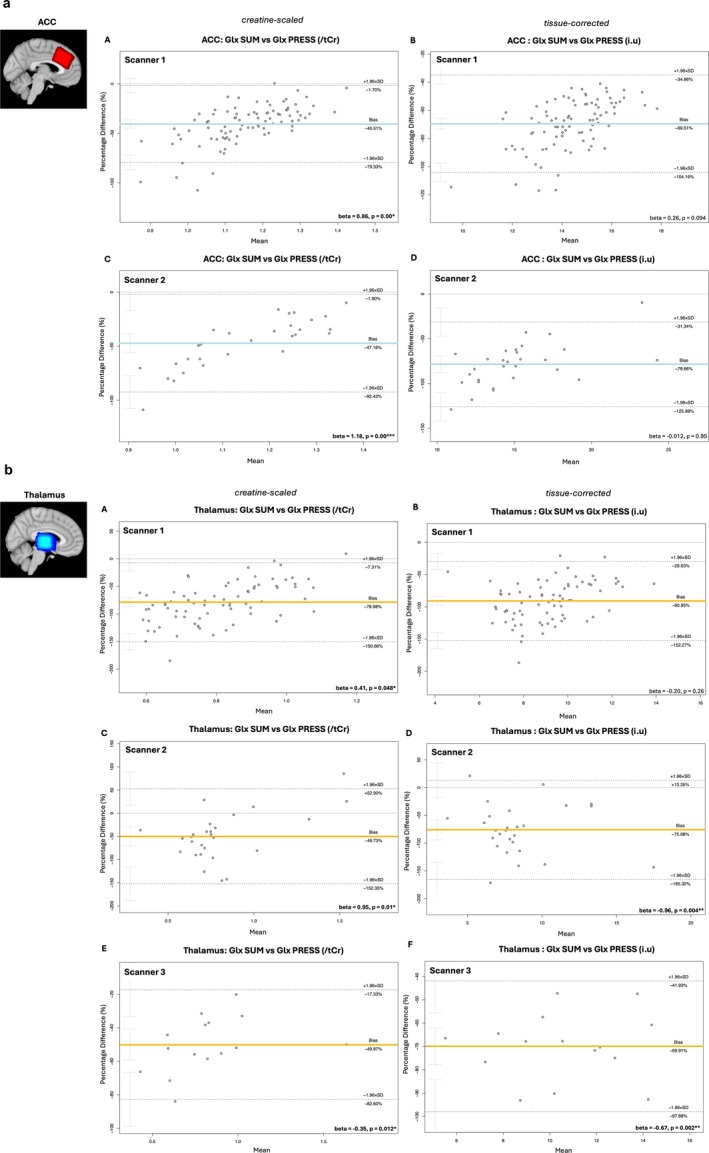
**(A) Bland–Altman (Giavarina version) plots comparing ACC Glx concentrations estimated from paired HERMES SUM and PRESS spectra per scanner.** For each plot, the percentage difference between paired measures is shown on the y‐axis, while the mean of the paired measures is shown the × axis. Solid blue lines represent the overall mean percentage difference (estimated bias) while dashed black lines represent the upper and lower limits of agreement (overall mean difference ± 1.96 standard deviation). Confidence intervals (95%) for limits of agreement are also shown. Linear regressions (model = percentage difference of paired measured ~ mean of paired measures) were used to identify proportional bias between paired measures, the resulting beta‐coefficient and corresponding *p* value is shown in the right‐hand corner of each plot; *p < 0.05, **p < 0.01, ***p < 0.001, ****p < 0.0001. **(B) Bland–Altman (Giavarina version) plots comparing thalamus Glx concentrations estimated from paired HERMES SUM and PRESS spectra per scanner.** For each plot, the percentage difference between paired measures is shown on the y‐axis, while the mean of the paired measures is shown the × axis. Solid yellow lines represent the overall mean percentage difference (estimated bias) while dashed black lines represent the upper and lower limits of agreement (overall mean difference ± 1.96 standard deviation). Confidence intervals (95%) for limits of agreement are also shown. Linear regressions (model = percentage difference of paired measured ~ mean of paired measures) were used to identify proportional bias between paired measures, the resulting beta‐coefficient and corresponding *p* value is shown in the right‐hand corner of each plot; *p < 0.05, **p < 0.01, ***p < 0.001, ****p < 0.0001.

Bland–Altman plots show significant bias between paired HERMES SUM and PRESS Glx estimates across all scanners, with SUM Glx estimates being systematically smaller than paired PRESS Glx estimates (for both the thalamus and ACC voxel per scanner; Figure 7A,B). The magnitude of systematic bias (mean percentage difference) was equivalent between tissue‐corrected and creatine‐scaled data for both voxels. Regression analysis found significant *proportional* bias between paired PRESS and HERMES SUM Glx estimates from the thalamus voxel of scanner 1 (β_creatine‐scaled_ = 0.41, *p* = 0.049, creatine‐scaled estimates only), the thalamus voxel of scanner 2 (β_creatine‐scaled_ = 0.95, *p* = 0.01; β_tissue‐corrected_ = −0.96, *p* = 0.04) and the thalamus voxel of scanner 3 (β_creatine‐scaled_ = −0.35, *p* = 0.012; β_tissue‐corrected_ = −0.67, *p* = 0.002). This was also true for the ACC voxel acquired by scanner 1 (β_creatine‐scaled_ = 0.86, *p* = 0.00) and scanner 2 (β_creatine‐scaled_ = 1.18, *p* = 0.00).

#### 3.3.3 Intraclass Correlation Coefficients Indicate Poor Consistency Between HERMES (GABA‐DIFF & SUM) and PRESS Glx Estimates

ICCs calculated from HERMES GABA‐DIFF and PRESS Glx estimates, and HERMES SUM and PRESS Glx estimates, were assessed per scanner (*step 3.3*). Results are presented in Supplementary Table S5. Consistency between ACC and thalamus HERMES GABA‐DIFF and PRESS Glx estimates (creatine‐scaled and tissue‐corrected) was poor across scanners. Consistency between thalamus and ACC HERMES SUM and PRESS Glx estimates was also poor across scanners. Note a few exceptions: for scanner 3, we observed moderate agreement between thalamus PRESS and HERMES SUM Glx estimates (ICC_creatine‐scaled_ = 0.86, ICC_tissue‐corrected_ = 0.66). For tissue‐corrected data from scanner 2, moderate consistency was observed between ACC HERMES SUM and PRESS Glx estimates (ICC_tissue‐corrected_ = 0.57).

### 3.4 Step 4. Variance of Glx Measurements per Spectra

#### 3.4.1 Coefficients of Variation

Intersubject CoVs were calculated for each Glx measurement (per scanner and per voxel; *step 4.1*). Results are reported in Table 3. For all scanners, intersubject CoVs calculated from HERMES SUM Glx estimates were greater than CoVs calculated from both HERMES GABA‐DIFF and PRESS Glx estimates from the ACC and thalamus. In general, PRESS Glx estimates had the smallest intersubject CoV across both voxels. The intersubject CoVs calculated from creatine‐scaled and tissue‐corrected Glx estimates from the thalamus were greater than those calculated from ACC; this was true for Glx estimates from all three spectra.

**TABLE 3:** Intersubject and mean intrasubject CoV calculated from HERMES (GABA‐DIFF & SUM) and PRESS Glx estimates.

	ACC (intersubject CoV)	Thalamus (intersubject CoV)	ACC (mean intrasubject CoV)	Thalamus (mean intrasubject CoV)
Scanner 1	Creatine‐scaled:	Creatine‐scaled:	Creatine‐scaled:	Creatine‐scaled:
	PRESS = 7.57	PRESS = 15.38	34.79	**59.31**
	DIFF = 16.57	DIFF = 28.14	Tissue‐corrected:	Tissue‐corrected:
	**SUM = 19.45**	**SUM = 42.81**	**36.60**	57.64
	Tissue‐corrected:	Tissue‐corrected:		
	PRESS = 8.33	PRESS = 17.43		
	DIFF = 14.32	DIFF = 25.42		
	**SUM = 20.22**	**SUM = 39.93**		
Scanner 2	Creatine scaled:	Creatine scaled:	Creatine‐scaled:	Creatine‐scaled:
	PRESS = 6.05	PRESS = 26.18	31.29	**69.91**
	DIFF = 10.07	DIFF = 41.61	Tissue‐corrected:	Tissue‐corrected:
	**SUM = 24.63**	**SUM = 70.25**	**39.24**	66.39
	Tissue‐corrected:	Tissue‐corrected:		
	PRESS = 17.32	PRESS = 40.54		
	DIFF = 19.53	DIFF = 35.59		
	**SUM = 38.12**	**SUM = 50.65**		
Scanner 3		Creatine‐scaled:		Creatine‐scaled:
		PRESS = 33.00		**51.91**
		DIFF = 25.45		Tissue‐corrected:
		**SUM = 38.39**		50.83
		Tissue‐corrected:		
		PRESS = 25.52		
		**DIFF = 29.61**		
		SUM = 28.41		

*Note:* Highlighted in bold per scanner, per voxel, is the Glx measurement with the highest intersubject/mean intrasubject CoV. Note that CoVs are expressed as a percentage.

Mean intrasubject CoVs are also reported in Table 3, with values ranging from 31.29% to 125%. Mean intrasubject CoVs calculated from creatine‐scaled data were generally greater than mean intrasubject CoVs calculated from tissue‐corrected data per scanner for the thalamus voxel; the opposite was true for the ACC voxel. Mean intrasubject CoVs calculated from thalamic Glx estimates were generally greater than mean intrasubject CoVs calculated from ACC Glx estimates per scanner.

### 3.5 Step 5. Effect of Diagnosis

Analyses were repeated per diagnostic group (typically developing and autism; *step* *5*). Note data from scanner 3 were excluded from this analysis due to an imbalance of participants per diagnostic group (Table 1). QM of MRS data and Glx concentrations are reported per diagnostic group in Supplementary Tables S6 and S7.

#### 3.5.1 Effect of Diagnostic Group: Paired Glx Estimates

Consistent with the main analysis, per diagnostic group (in data from scanners 1 and 2), HERMES GABA‐DIFF Glx estimates were greater than paired PRESS Glx estimates, while HERMES SUM Glx estimates were smaller than paired PRESS Glx estimates (in the same participant). This was generally true for both the thalamus and ACC voxels (see Supplementary Figure S6 for specific significant differences per scanner).

#### 3.5.2 Effect of Diagnosis: Spearman Partial Correlation Coefficients

When data was grouped by diagnosis, in TD data, we observed no significant correlations between HERMES (SUM and GABA‐DIFF) and PRESS Glx estimates. In autism data, a significant positive partial correlation (controlling for FWHM) was observed between ACC Glx estimates from HERMES GABA‐DIFF and PRESS acquired by scanner 1 (ρ_creatine‐scaled_ = 0.45, *p*_adjusted_ = 0.004; creatine‐scaled only). No additional significant correlations were observed.

#### 3.5.3 Effect of Diagnosis: Bland–Altman Analysis

Results per diagnostic group for Bland–Altman analysis are largely consistent with those outlined in the main pooled analysis (Supplementary Figures S7–S14 and Supplementary Table S8). Bland–Altman shows that for autistic and TD participants, HERMES GABA‐DIFF Glx estimates were systematically greater than paired PRESS Glx estimates (creatine‐scaled and tissue‐corrected). Conversely, HERMES SUM Glx estimates were systematically smaller than paired PRESS Glx estimates (tissue‐corrected and creatine‐scaled data). This was true for the ACC and thalamus voxels (Supplementary Figure S7–S14). Significant positive *proportional* bias was also observed between paired PRESS and HERMES GABA‐DIFF Glx estimates and between paired PRESS and HERMES SUM Glx estimates per diagnostic group (Supplementary Table S8). Note that for both TD and autism groups, proportional bias was more evident in creatine‐scaled data compared to tissue‐corrected.

#### 3.5.4 Effect of Diagnosis: ICC Values

ICC values, calculated between HERMES (SUM and GABA‐DIFF) and PRESS Glx estimates per scanner per diagnosis (Supplementary Table S9), are consistent with the main analysis in that the agreement between HERMES GABA‐DIFF and PRESS, and HERMES SUM and PRESS was generally poor. There are a few exceptions to this: we observed moderate agreement between thalamic HERMES SUM and PRESS creatine‐scaled Glx estimates in TD data from scanner 2 (ICC_thalamus_ = 0.64). There was also moderate agreement between ACC HERMES SUM and PRESS tissue‐corrected Glx estimates; this was true in autism and TD data from scanner 2 (ICC_autism_ = 0.63, ICC_TD_ = 0.56). Finally, we also observed moderate agreement between thalamic tissue‐corrected HERMES SUM and PRESS Glx estimates in TD data from scanner 2 (ICC = 0.66).

#### 3.5.5 Effect of Diagnosis: Intersubject and Intrasubject CoVs

Results per diagnosis reflect the main diagnostic pooled analyses (Supplementary Table S10). In general, per diagnostic group (per scanner 1 and 2), PRESS Glx estimates had the smallest intersubject CoVs, while HERMES SUM Glx estimates had the largest intersubject CoVs. Per scanner and per diagnostic group, the intersubject CoVs of Glx estimates from the thalamus were greater than the CoVs of Glx estimates from the ACC voxel. Mean intrasubject CoVs ranged from 35%–72% for autism cohorts (from scanner 1 and scanner 2; Supplementary Table S9) and 27%–64% for TD cohorts (from scanner 1 and scanner 2).

## 4 Discussion

In this paper, we have evaluated the agreement between Glx estimates derived from HERMES GABA‐DIFF, HERMES SUM and PRESS spectra acquired from a large sample of typically developing and autistic preschoolers, adolescents and adults and assessed any additional effects related to brain region, scanner, demographic factors and quantification approach.

Our findings reveal poor agreement between Glx estimates from HERMES GABA‐DIFF and short‐TE PRESS, a trend consistent across scanners, thalamus and ACC voxels, ages and diagnostic groups (TD and autism). We observe poor consistency (low ICC, poor correlation and proportional biases) and also poor concordance, meaning that the values not only differ in magnitude but also do not preserve individual rank or scale predictably across methods. HERMES SUM Glx showed slightly improved correlation and agreement with short‐TE PRESS Glx, specifically in preschooler cohorts; however, this was not reliable or consistent across scanners or quantification approaches. In line with this generally poor agreement, the mean intrasubject variability (CoV) of paired Glx measurements across different spectra in the same participant (HERMES GABA‐DIFF, HERMES SUM and PRESS) ranged from 27% to 135%, substantially higher than the ~10% typically reported for repeated metabolite estimates using the same sequence in the same participant [66, 67]. This further suggests that HERMES (GABA‐DIFF & SUM) and PRESS Glx measurements are not equivalent.

When considering specific differences between Glx estimates, generally consistent across scanners, voxels, age groups and diagnostic groups, were findings that HERMES GABA‐DIFF Glx estimates were systematically greater than PRESS Glx estimates, while HERMES SUM Glx estimates were systematically lower than PRESS Glx estimates. Significant proportional differences between HERMES (GABA‐DIFF & SUM) and PRESS Glx measurements were also identified, with the discrepancy between Glx estimates generally increasing with Glx magnitude. Because we focused on assessing the consistency of Glx measurements in our agreement analyses (rather than the concordance between raw Glx values), these proportional differences likely drive the poor agreement observed between HERMES and PRESS Glx (poor correlation & poor ICC values), as opposed to the linear scaling between estimates (i.e., systematic differences that indicate consistent overestimation or underestimation). Proportional biases are especially important to identify because, unlike systematic differences, they cannot be easily corrected for (as the difference between measures is dependent on Glx magnitude) and thus pose a significant challenge for comparing and integrating Glx data across different acquisition methods in multisite settings and between different studies.

Overall, our findings suggest that Glx estimates from HERMES SUM and GABA‐DIFF spectra do not substitute for those derived from short‐TE PRESS. This is consistent with findings for GABA‐edited MEGA‐PRESS Glx [30, 31, 42], although we additionally show that biases are generally consistent across different scanners, diagnostic groups and across creatine‐scaled and tissue‐corrected data. We discuss this and the factors contributing to the poor agreement between HERMES and PRESS Glx in more detail below.

### 4.1 Contribution of Data Quality

Data quality differences likely contribute to differences in Glx estimates obtained from HERMES compared to Glx estimates obtained from PRESS. For example, across scanners, age and diagnostic groups, the consistency between HERMES (GABA‐DIFF and SUM) and PRESS Glx estimates was greater in data acquired from the ACC compared to the thalamus (note, however, that consistency was still poor to moderate). As opposed to a regional effect, this discrepancy likely reflects differences in spectral quality, as thalamic PRESS and HERMES spectra showed poorer data quality compared to ACC spectra, characterised by lower SNR and greater linewidth. Glx measurements from the thalamus thus exhibited increased nuisance variance across participants (evidenced by higher intersubject CoV) and within repeated measurements per participant (evidenced by higher intrasubject CoV) compared to data from the ACC. The thalamus's deep midbrain location (associated with poorer shimming) likely contributes to the additional ‘noise’ within thalamic Glx measurements, impairing consistency between paired HERMES and PRESS Glx estimates when compared to the ACC [68, 69]. Data quality differences likely also contribute to differing Glx estimate consistency between scanners. Data from scanners 1 and 3 were collected at the same site, and while QM between these two scanners did significantly differ, these scanners had the noticeably smaller intersubject and intrasubject CoV compared to scanner 2. This reduced noise within Glx measurements is potentially a result of differences in voxel placement and shimming practices between scanners (and sites) and likely explains why correlations between PRESS and HERMES Glx were stronger in data from scanners 1 and 3 compared to 2.

HERMES acquisitions have a long echo time and a four‐step acquisition scheme, which makes HERMES particularly sensitive to motion and scanner instabilities [70]. Accordingly, HERMES SUM Glx estimates were the ‘nosiest’ (highest intersubject CoVs) of the measurements evaluated, even compared to HERMES GABA‐DIFF. This suggests that the summation process amplifies noise compared to subtraction for the GABA‐DIFF spectra. Furthermore, noise levels in the SUM spectra (as indexed by FWHM) were significantly correlated with Glx quantification, which we controlled for where possible and still observed poor consistency between Glx estimates. These findings highlight that data quality can influence Glx estimation, even when acquisitions are performed within the same session and participant, underscoring the importance of adhering to rigorous and consistent acquisition standards when collecting MRS data. Importantly, however, differences in the quality of HERMES and PRESS spectra were inconsistent between scanners and voxels and were not always significant. Thus, while they likely contribute, these data quality effects *alone* cannot explain the consistent systematic and proportional differences between Glx estimates identified. In addition, there are potentially real differences in the underlying Glx quantities measured per sequence.

### 4.2 Differences in Glx Quantities Measured and Modelled

The Glx signal measured across PRESS, HERMES SUM and HERMES GABA‐DIFF acquisitions likely differs due to a combination of T2 relaxation, J‐modulation, and sequence‐specific acquisition properties. In particular, glutamine and macromolecule signals are suppressed at echo times between 40‐80 ms [34, 36, 71], and as such, Glx resonances obtained from long‐TE PRESS (TE = 80 ms) resemble phantom glutamate only spectra [34, 42]. Macromolecule basis functions and TE‐specific basis sets were used during Glx quantification to mitigate these effects. While such modelling helps reduce overestimation of Glx at short‐TEs [72] and account for TE‐specific differences in glutamine contribution to the Glx signal due to J‐modulation [60], these functions are still only approximations of in vivo spectrums and are relatively poorly characterised per spectra/sequence [31, 55, 60]. Similarly, we applied post hoc Glx signal T2 relaxation corrections using literature‐derived values [55]. Because metabolite T2 relaxation times differ per scanner and per age group, this approach may fail to appropriately account for increased T2 relaxation of both glutamate and glutamine at long‐TEs in HERMES compared to PRESS [73]. Inconsistent with Glx signal T2 relaxation and J‐modulation effects, however, we find HERMES GABA‐DIFF Glx estimates were generally systematically larger than those derived from short‐TE PRESS. This contradicts the expectation that short‐TE PRESS acquisitions, where macromolecule and glutamine contributions are greater, would yield greater Glx values compared to long‐TE HERMES. Our findings also differ from Bell et al., 2020 [42], who found that MEGA‐PRESS (TE = 68 ms) GABA‐DIFF spectra produce systematically lower Glx estimates compared to short‐TE PRESS (TE = 35 ms), as would be expected.

Basis set influences, as well as differences in MRS processing software, may explain the discrepancies between our findings. For preprocessing, fitting and quantification of MRS data, we used Osprey (version 2022a), chosen due to its open‐source nature and advanced fitting approaches compared to older software [55]. In particular, Osprey can perform batch processing using the most advanced postprocessing, co‐registration and quantification approaches currently available. Furthermore, Osprey allows for fitting of both the HERMES SUM and HERMES GABA‐DIFF spectra within a single analysis. However, it is important to acknowledge that Osprey is not a validated gold standard for Glx quantification. Osprey Glx (PRESS TE = 35 ms) estimates have shown poor agreement with Glx estimates obtained using closed‐source MRS processing software such as LCModel, which was employed by Bell et al. 2020 [42]. Furthermore, it has been demonstrated that Glx estimates from identical data analysed in Gannet, the predecessor to Osprey, and LCModel show only modest correlation [31]. As well as the chosen software and fitting algorithm, basis sets can significantly influence metabolite quantification, even within the same processing tool [29]. Because the basis sets used were simulated based on scanner parameters, specific pulse sequences and TEs [55], a degree of bias is inevitable when modelling Glx in spectra acquired with different sequence parameters [30]. The simulated basis sets used also assume optimal scan conditions, while B_0_ inhomogeneities, pulse frequency selectivity, spin system parameters and relaxation effects likely vary per spectra, per scanner and *per voxel*, further biasing Glx estimates. Future work should investigate whether our findings hold across different MRS analysis pipelines to ensure transferability and explore this apparent inconsistency in the bias between Glx estimates from HERMES GABA‐DIFF spectra and MEGA‐PRESS GABA‐DIFF spectra compared to short‐TE PRESS.

Note glutamate and glutamine only estimates were not reported on in this study as they are poorly resolved at 3 T (due to poor chemical shift dispersion). Thus measurement of scanner‐specific metabolite J‐modulation effects, as well as macromolecule baselines using adiabatic pulse inversion recovery experiments [74] and/or incorporation of a co‐editing macromolecule ‘nulling’ pulse into the GABA_edit‐off_ editing scheme [2] would be necessary to validate if Glx signal composition differences between short and long TEs underlie the observed discrepancies in Glx estimates from PRESS and HERMES. In this study, these measurements were not performed due to limitations on scan duration, as well as the potential increases in frequency drift (due to the narrower editing pulses required for macromolecule nulling [2, 75]) and motion artefacts (due to noninterleaved transients [76]). Furthermore, we opted to examine Glx given its consistent reporting in the literature across both edited and nonedited sequences, ensuring the broader relevance of this work.

### 4.3 Scanner Instabilities

The *proportional* bias observed between HERMES Glx estimates and short‐TE PRESS Glx potentially reflects B_0_ drift during scanning, which affects the accuracy of Glx quantification from HERMES GABA‐DIFF [34, 77]. Measurement of Glx from HERMES GABA‐DIFF spectra relies on the precise co‐editing of Glx coupling partners at 2.1 ppm by the frequency‐selective 1.9‐ppm GABA‐editing pulse. B_0_ drift across the scanning period (due to heating and scanner instabilities) alters the precision of the 1.9‐ppm editing pulse, altering the degree of interaction with glutamate and glutamine coupling partners at 2.1 ppm and so the efficacy of Glx co‐editing across the scan period [34, 77]. This has been shown to alter the Glx signal amplitude and so Glx quantification from edited spectra (MEGA‐PRESS), with a downfield frequency offset of the GABA‐editing pulse (towards the 2.1 ppm) shown to significantly positively correlate with glutamate concentrations [77]. Furthermore, differences in the efficacy of glutamine co‐editing compared to glutamate co‐editing (glutamine resonances being slightly further from 1.9 ppm) could result in differing contributions of their signals to co‐edited Glx [42].

The narrow bandwidth and long duration of the HERMES editing pulses (20 ms) make HERMES spectra particularly susceptible to B_0_ drift (as does the longer scan duration), more so than MEGA‐PRESS [2, 43, 76]. PRESS spectra, which contain unedited metabolite signals, are less susceptible to such frequency drift effects, potentially contributing to the proportional discrepancy between PRESS and HERMES GABA‐DIFF Glx estimates [30]. Note, however, that random fluctuations in editing efficiency during a scanning session would not be expected to produce a consistent proportional bias, as such variations may cancel out over the course of the acquisition. Therefore, other factors (including those discussed above) likely also contribute to the proportional bias between Glx estimates observed.

Finally note that, despite employing a universal HERMES acquisition across scanners, HERMES Glx concentrations were significantly different across scanners. While differing sample characteristics per scanner may contribute (diagnosis and age), this suggests that harmonisation approaches or statistical controls are still necessary when integrating Glx concentrations acquired from multiple sites with ‘universal’ sequences.

### 4.4 Effect of Demographic Factors

While our cohort was predominantly comprised of participants with autism, subgroup analyses indicated that the observed trends were consistent across both autistic and typically developing groups. Given the disparity in participant numbers per diagnostic group, it is still possible, however, that our findings are population‐specific, particularly due to potential differences in Glx signal T2 relaxation effects in autistic and TD populations. Differing Glx signal T2 effects likely also influence our age‐group analysis, with known differences in metabolite T2 relaxation across age [78]. HERMES SUM and PRESS Glx estimates acquired from preschoolers (from scanner 3) did show better agreement compared to adult data from scanners 1 and 2 (and this was significant when comparing HERMES SUM and PRESS Glx correlations between scanners). However, because we are comparing data from scanner 3, which exclusively scanned preschoolers, with data from scanners 1 and 2, which exclusively scanned adolescents and adults, it is difficult to determine whether the observed differences are due to site effects or true age‐related effects. Furthermore, scans of preschoolers were conducted during sleep (and adult scans were not), which has potential implications for data quality (e.g., reducing motion), which, as outlined above, is also associated with consistency between Glx estimates. Given this, the findings in this study are likely most applicable to adults and adolescents, as these comprised the majority of our sample, and the data trends observed were consistent across two scanners assessing this population.

Note that we found similar intersubject and intrasubject CoVs for Glx estimates across spectra, voxels and scanners for both age groups and diagnostic groups, suggesting that the autism data and the paediatric (preschooler) data included did not contain additional nuisance variance. This is an important consideration, given previous research suggests that autistic individuals and children may move more during scanning [45, 79]. Given that the data exclusion rate was also comparable between diagnostic and age groups, we do not consider this a challenge in this study, potentially because preschooler scans were performed during sleep.

### 4.5 Limitations

Short‐TE PRESS Glx was used as a reference, but we have no ground truth for Glx quantification; furthermore, other approaches may be more suitable depending on the research question. For example, as previously discussed, long‐TE PRESS (TE = 80 ms) has been shown to be suitable for glutamate‐only measurements [34], along with TE‐averaged PRESS [32, 35, 36, 46] or 2D‐JPRESS [30, 33]. However, short‐TE PRESS is widely used, repeatable [35, 42, 50], facilitates the quantification of multiple metabolites (not just Glx), has a short duration and has established and open‐access processing pipelines. It has also previously been used to assess the reliability of MEGA‐PRESS Glx [31, 42]. Thus, it was a suitable standard for these analyses. Furthermore, a definitive *ground truth* may not be applicable to in vivo quantification, given the complex and variable nature of the underlying biology and measurement conditions.

## 5 Conclusions

We show that there is significant systematic and proportional bias between Glx estimates from HERMES (SUM and GABA‐DIFF) and short‐TE PRESS spectra, and so poor agreement. This was consistent across datasets from three scanners, two voxels (thalamic and ACC), two age groups (preschoolers and adolescents/adults) and two diagnostic groups (typically developing and autistic cohorts). Glx estimates from HERMES (SUM and GABA‐DIFF) are thus not consistent with those from short‐TE PRESS, and this discrepancy persists across biological, technical and demographic variability, conditions reflective of realistic, multisite study designs. The continued use of PRESS Glx is thus recommended, ensuring consistency and so the reliable interpretation of Glx measurements across studies. Our results highlight the importance of sequence selection and the need for careful consideration when integrating and interpreting data across different acquisitions.

## Author Contributions

**Alice Thomson:** writing – original draft, investigation, methodology, formal analysis, data curation, visualization. **Viola Hollestein:** writing – review and editing, methodology, data curation. **Amy Goodwin:** writing – review and editing, validation, project administration. **Anne Fritz:** writing – review and editing, data curation, validation, project administration. **Beth Oakley:** writing – review and editing, validation, project administration. **Declan Murphy:** writing – review and editing, validation, project administration, funding acquisition. **Edward Bullock:** writing – review and editing, data curation, project administration. **Ellen Demurie:** writing – review and editing, project administration. **Eva Loth:** writing – review and editing, validation, project administration, funding acquisition. **Giorgia Bussu:** writing – review and editing, validation, project administration. **Herbert Roeyers:** writing – review and editing, validation, project administration, funding acquisition. **Isabel Yorke:** writing – review and editing, validation. **Jan K. Buitelaar:** writing – review and editing, validation, project administration, funding acquisition. **Julia Koziel:** writing – review and editing, validation, project administration. **Laura Colomar:** writing – review and editing, validation, project administration. **Manon A. Krol:** writing – review and editing, validation, project administration. **Matthew Bowdler:** writing – review and editing, validation, project administration. **Nele Herregods:** writing – review and editing, validation, project administration. **Pascal Aggensteiner:** writing – review and editing, validation, project administration, funding acquisition. **Pim Pullens:** writing – review and editing, validation, project administration, funding acquisition. **Rosemary Holt:** writing – review and editing, validation, project administration, funding acquisition. **Terje Falck‐Ytter:** writing – review and editing, validation, project administration, funding acquisition. **Tony Charman:** writing – review and editing, validation, project administration, funding acquisition. **Tomoki Arichi:** writing – review and editing, validation, funding acquisition, supervision. **Nicolaas A. Puts:** writing – review and editing, data curation, validation, methodology, software funding acquisition, supervision.

## Funding

This work was supported by Innovative Medicines Initiative 2 Joint Undertaking, 777394; Medical Research Council, 10.13039/501100000265, MR/Y009665/1, MR/N026063/1; and Horizon2020, 847818.

## Conflicts of Interest

The authors declare no conflicts of interest.

## Supporting information


**Table S1:** Sequence acquisition parameters per scanner.
**Table S2:** Data excluded per scanner and why.
**Table S3:** Quality of thalamus and ACC PRESS and HERMES spectra per scanner. Differences in the quality of MRS data per scanner were assessed using Kruskal–Wallis tests, the calculated H statistics and corresponding p values are displayed in the table, with padjusted < 0.05 indicating a significant difference in the quality of MRS data per scanner after Bonferroni correction. Note SNR: signal to noise ratio of total creatine and FWHM: full width half maximum of total creatine signal.
**Figure S1:** Mean HERMES GABA‐DIFF, HERMES SUM and PRESS spectra per scanner, showing model fit, model baseline and median residual fits (error bars).
**Table S4:** Significant correlations between FWHM and Glx estimates from HERMES SUM, HERMES GABA‐DIFF and PRESS spectra. Correlations between FWHM and Glx estimates was assessed using Spearman's Rank correlation coefficients. Correlation coefficients are displayed in the table, with *p*_adjusted_ < 0.05 indicating significance after Bonferroni correction. Note we chose to only observe correlations between Glx and FWHM, as QM are colinear, and thus controlling for one in subsequent analysis is sufficient. Only significant correlations are reported.
**Figure S2:** Quality of paired HERMES (GABA‐DIFF & SUM) and PRESS spectra acquired from the thalamus voxel significantly differs. Friedman one‐way repeated measure analysis was used to assess if there were significant differences in the quality of spectra (HERMES GABA‐DIFF (DIFF), HERMES SUM (SUM) and PRESS) acquired from the same voxel (in the same participant). Pairwise Wilcoxon rank sum tests were used for post hoc testing to isolate specific differences in quality metrics between paired spectra, with Bonferroni correction for multiple comparisons. Results of post hoc testing are shown for the thalamus voxel, with p values adjusted for multiple‐comparisons using Bonferroni correction. Ns: Nonsignificant, *padjusted < 0.05, ** padjusted < 0.01, *** padjusted < 0.001, **** padjusted < 0.0001, SNR: signal to noise ratio of total creatine, FWHM: full width half maximum of total creatine.
**Figure S3:** Quality of paired HERMES (GABA‐DIFF & SUM) and PRESS spectra acquired from the ACC voxel significantly differs. Friedman one‐way repeated measure analysis was used to assess if there were significant differences in the quality of spectra (HERMES GABA‐DIFF (DIFF), HERMES SUM (SUM) and PRESS) acquired from the same voxel (in the same participant). Pairwise Wilcoxon Rank sum tests were used for post hoc testing to isolate specific differences in quality metrics between paired spectra, with Bonferroni correction for multiple comparisons. Results of post hoc testing are shown for the ACC voxel, with p values adjusted for multiple‐comparisons using Bonferroni correction. Ns: Nonsignificant, *padjusted < 0.05, ** padjusted < 0.01, *** padjusted < 0.001, **** padjusted < 0.0001, SNR: signal to noise ratio of total creatine, FWHM: full width half maximum of total creatine.
**Figure S4:** The quality of MRS data (HERMES (GABA‐DIFF & SUM) and PRESS) acquired from the same participant differs between thalamus and ACC voxels. Friedman one‐way repeated measure analyses were used to assess if quality of MRS data (HERMES (DIFF & SUM) and PRESS) acquired from the same participant significantly differed between voxels (thalamus and ACC). Pairwise Wilcoxon Rank sum tests were used for post hoc testing where appropriate, with Bonferroni correction for multiple comparisons. Results of post hoc testing are shown, with p values adjusted for multiple‐comparisons using Bonferroni correction. Ns: Nonsignificant, *padjusted < 0.05, ** padjusted < 0.01, *** padjusted < 0.001, **** padjusted < 0.0001. Note scanner 4 did not record data from an ACC voxel.
**Figure S5:** Glx (creatine‐scaled [/tCr] and tissue‐corrected [i.u]) concentrations quantified from the PRESS, HERMES GABA‐DIFF (DIFF) and HERMES SUM spectra per scanner for the ACC and thalamus voxels. Kruskal–Wallis tests were used to assess if PRESS, HERMES DIFF and HERMES SUM estimated Glx concentrations differed between scanners 1–3 for the thalamus and ACC voxels. Mann–Whitney–Wilcoxon tests were used for post hoc testing to isolate specific differences between scanners where appropriate, with Bonferroni correction for multiple comparisons. Results of post hoc testing are shown. Ns: Nonsignificant, *p < 0.05, **p < 0.01, ***p < 0.001, ****p < 0.0001. Note scanner 3 did not record data from an ACC voxel.
**Table S5:** ICC values (and 95% confidence intervals) calculated between HERMES (GABA‐DIFF and SUM) and PRESS Glx estimates per voxel per scanner for tissue‐corrected and creatine‐scaled data. Note overall refers to data pooled across voxels.
**Table S6:** Quality of thalamus and ACC PRESS and HERMES spectra per scanner per diagnosis. Differences in the quality of MRS data per scanner were assessed using Kruskal–Wallis tests, the calculated H statistics and corresponding p values are displayed in the table, with p < 0.05 indicating a significant difference in the quality of MRS data per scanner. Note SNR: signal to noise ratio of total creatine and FWHM: full width half maximum of total creatine.
**Table S7:** Glx concentrations quantified from the thalamus and ACC PRESS and HERMES spectra per scanner per diagnosis.
**Figure S6:** Thalamus and ACC Glx concentrations (creatine‐scaled (/tCr) and tissue‐corrected [i.u]) estimated from the HERMES DIFF, HERMES SUM and PRESS spectra per scanner per diagnostic group. Tissue corrected (i.u) and creatine‐scaled (/tCr) Glx concentrations estimated from the HERMES DIFF (DIFF), HERMES SUM (SUM) and PRESS spectra are shown per voxel, per scanner and per diagnostic group (TD and autism). Friedman one‐way repeated measure analysis was used to assess if there were significant differences in the concentration of Glx estimated from the HERMES DIFF (DIFF), HERMES SUM (SUM) and PRESS spectra acquired from the same voxel per diagnostic group. Pairwise Wilcoxon rank sum tests were used for post hoc testing where appropriate to isolate specific differences in Glx concentration between paired spectra, with Bonferroni correction for multiple comparisons. Results of post hoc testing are shown per voxel.
**Figure S7:** Bland–Altman (Giavarina version) plots comparing Glx concentrations estimated from paired HERMES GABA‐DIFF (Glx DIFF) and PRESS spectra acquired from a thalamus voxel on scanner 1 for autism and TD groups Bland–Altman (Giavarina version) plots comparing creatine‐scaled (/tCr) and tissue‐corrected (i.u) Glx estimated from paired HERMES DIFF and PRESS spectra from the thalamus voxel, scanner 1, for autism and TD groups. For each plot, the percentage difference between paired measures is shown on the y‐axis, while the mean of the paired measures is shown the x axis. Dashed lines represent the overall mean percentage difference (estimated bias), the upper and lower limits of agreement (overall mean difference ±1.96 standard deviation). Confidence intervals for limits of agreement are also shown. Linear regressions (model = percentage difference of paired measured ~ mean of paired measures) were used to identify proportional bias between paired measures, the resulting beta coefficient and corresponding p value is shown in the right‐hand corner of each plot.
**Figure S8:** Bland–Altman (Giavarina version) plots comparing Glx concentrations estimated from paired HERMES GABA‐DIFF and PRESS spectra acquired from a ACC voxel on scanner 1 for autism and TD groups. Bland–Altman (Giavarina version) plots comparing creatine‐scaled (/tCr) and tissue‐corrected (i.u) Glx estimated from paired HERMES DIFF and PRESS spectra from the ACC voxel, scanner 1, for autism and TD groups. For each plot, the percentage difference between paired measures is shown on the y‐axis, while the mean of the paired measures is shown the x axis. Dashed lines represent the overall mean percentage difference (estimated bias), the upper and lower limits of agreement (overall mean difference ±1.96 standard deviation). Confidence intervals for limits of agreement are also shown. Linear regressions (model = percentage difference of paired measured ~ mean of paired measures) were used to identify proportional bias between paired measures, the resulting beta coefficient and corresponding p value is shown in the right‐hand corner of each plot.
**Figure S9:** Bland–Altman (Giavarina version) plots comparing Glx concentrations estimated from paired HERMES DIFF and PRESS spectra acquired from a thalamus voxel on scanner 2 for autism and TD groups. Bland–Altman (Giavarina version) plots comparing creatine‐scaled (/tCr) and tissue‐corrected (i.u) Glx estimated from paired HERMES DIFF and PRESS spectra from the thalamus voxel, scanner 2, for autism and TD groups. For each plot, the percentage difference between paired measures is shown on the y‐axis, while the mean of the paired measures is shown the x axis. Dashed lines represent the overall mean percentage difference (estimated bias), the upper and lower limits of agreement (overall mean difference ±1.96 standard deviation). Confidence intervals for limits of agreement are also shown. Linear regressions (model = percentage difference of paired measured ~ mean of paired measures) were used to identify proportional bias between paired measures, the resulting beta coefficient and corresponding p value is shown in the right‐hand corner of each plot.
**Figure S10:** Bland–Altman (Giavarina version) plots comparing Glx concentrations estimated from paired HERMES GABA‐DIFF and PRESS spectra acquired from an ACC voxel on scanner 2 for autism and TD groups. Bland–Altman (Giavarina version) plots comparing creatine‐scaled (/tCr) and tissue‐corrected (i.u) Glx estimated from paired HERMES DIFF and PRESS spectra from the ACC voxel, scanner 2, for autism and TD groups. For each plot, the percentage difference between paired measures is shown on the y‐axis, while the mean of the paired measures is shown the x axis. Dashed lines represent the overall mean percentage difference (estimated bias), the upper and lower limits of agreement (overall mean difference ±1.96 standard deviation). Confidence intervals for limits of agreement are also shown. Linear regressions (model = percentage difference of paired measured ~ mean of paired measures) were used to identify proportional bias between paired measures, the resulting beta coefficient and corresponding p value is shown in the right‐hand corner of each plot.
**Figure S11:** Bland–Altman (Giavarina version) plots comparing Glx concentrations estimated from paired HERMES SUM and PRESS spectra acquired from a thalamus voxel on scanner 1 for autism and TD groups. Bland–Altman (Giavarina version) plots comparing creatine‐scaled (/tCr) and tissue‐corrected (i.u) Glx estimated from paired HERMES SUM and PRESS spectra from the thalamus voxel, scanner 1, for autism and TD groups. For each plot, the percentage difference between paired measures is shown on the y‐axis, while the mean of the paired measures is shown the x axis. Dashed lines represent the overall mean percentage difference (estimated bias), the upper and lower limits of agreement (overall mean difference ±1.96 standard deviation). Confidence intervals for limits of agreement are also shown. Linear regressions (model = percentage difference of paired measured ~ mean of paired measures) were used to identify proportional bias between paired measures, the resulting beta coefficient and corresponding p value is shown in the right‐hand corner of each plot.
**Figure S12:** Bland–Altman (Giavarina version) plots comparing Glx concentrations estimated from paired HERMES SUM and PRESS spectra acquired from an ACC voxel on scanner 1 for autism and TD groups. Bland–Altman (Giavarina version) plots comparing creatine‐scaled (/tCr) and tissue‐corrected (i.u) Glx estimated from paired HERMES SUM and PRESS spectra from the ACC voxel, scanner 1, for autism and TD groups. For each plot, the percentage difference between paired measures is shown on the y‐axis, while the mean of the paired measures is shown the x axis. Dashed lines represent the overall mean percentage difference (estimated bias), the upper and lower limits of agreement (overall mean difference ±1.96 standard deviation). Confidence intervals for limits of agreement are also shown. Linear regressions (model = percentage difference of paired measured ~ mean of paired measures) were used to identify proportional bias between paired measures, the resulting beta coefficient and corresponding p value is shown in the right‐hand corner of each plot.
**Figure S13:** Bland–Altman (Giavarina version) plots comparing Glx concentrations estimated from paired HERMES SUM and PRESS spectra acquired from a thalamus voxel on scanner 2 for autism and TD groups. Chnag 2 Bland–Altman (Giavarina version) plots comparing creatine‐scaled (/tCr) and tissue‐corrected (i.u) Glx estimated from paired HERMES SUM and PRESS spectra from the thalamus voxel, scanner 2, for autism and TD groups. For each plot, the percentage difference between paired measures is shown on the y‐axis, while the mean of the paired measures is shown the x‐axis. Dashed lines represent the overall mean percentage difference (estimated bias), the upper and lower limits of agreement (overall mean difference ±1.96 standard deviation). Confidence intervals for limits of agreement are also shown. Linear regressions (model = percentage difference of paired measured ~ mean of paired measures) were used to identify proportional bias between paired measures, the resulting beta coefficient and corresponding p value is shown in the right‐hand corner of each plot.
**Figure S14:** Bland–Altman (Giavarina version) plots comparing Glx concentrations estimated from paired HERMES SUM and PRESS spectra acquired from an ACC voxel on scanner 2 for autism and TD groups. Bland–Altman (Giavarina version) plots comparing creatine‐scaled (/tCr) and tissue‐corrected (i.u) Glx estimated from paired HERMES SUM and PRESS spectra from the ACC voxel, scanner 2, for autism and TD groups. For each plot, the percentage difference between paired measures is shown on the y‐axis, while the mean of the paired measures is shown the x‐axis. Dashed lines represent the overall mean percentage difference (estimated bias), the upper and lower limits of agreement (overall mean difference ±1.96 standard deviation). Confidence intervals for limits of agreement are also shown. Linear regressions (model = percentage difference of paired measured ~ mean of paired measures) were used to identify proportional bias between paired measures, the resulting beta coefficient and corresponding p value is shown in the right‐hand corner of each plot.
**Table S8:** Bland–Altman regression analysis by diagnosis. The table shows the results from linear regressions (model = percentage difference of paired measured ~ mean of paired measures) used to identify proportional bias between paired measures. Significant beta coefficients indicate significant proportional bias (as the magnitude of Glx changes, the disparity between measurements changes). NS = nonsignificant proportional bias.
**Table S9:** ICC values calculated between HERMES (DIFF and SUM) and PRESS Glx estimates per voxel per scanner for tissue‐corrected and creatine‐scaled data per diagnostic group.
**Table S10:** Intersubject CoV of Glx estimates from HERMES (DIFF and SUM) and PRESS Glx spectra per scanner for tissue‐corrected and creatine‐scaled data from the thalamus and ACC, per diagnostic group.

## Data Availability

Osprey 2.4.0 is available through: https://github.com/schorschinho/osprey The data that support the findings of this study are available on request from the corresponding author. The data are not publicly available due to privacy or ethical restrictions.
