# Supplementary material for: In Vivo Glx Measurements From GABA‐Edited HERMES at 3 T Are Not Consistent With Those From Short‐TE PRESS Across Scanners, Brain Regions, Diagnostic and Age Groups
Source: NMR Biomed. 2025 Dec 3;39(1):e70171. doi: 10.1002/nbm.70171 (PMC12673632; doi:10.1002/nbm.70171)
Supplement: Supplementary file 1 — Table S1: Sequence acquisition parameters per scanner. Table S2: Data excluded per scanner and why. Table S3: Quality of thalamus and ACC PRESS and HERMES spectra per scanner. Differences in the quality of MRS data per scanner were assessed using Kruskal–Wallis tests, the calculated H statistics and corresponding p values are displayed in the table, with padjusted < 0.05 indicating a significant difference in the quality of MRS data per scanner after Bonferroni correction. Note SNR: signal to noise ratio of total creatine and FWHM: full width half maximum of total creatine signal. Figure S1: Mean HERMES GABA‐DIFF, HERMES SUM and PRESS spectra per scanner, showing model fit, model baseline and median residual fits (error bars). Table S4: Significant correlations between FWHM and Glx estimates from HERMES SUM, HERMES GABA‐DIFF and PRESS spectra. Correlations between FWHM and Glx estimates was assessed using Spearman's Rank correlation coefficients. Correlation coefficients are displayed in the table, with p adjusted < 0.05 indicating significance after Bonferroni correction. Note we chose to only observe correlations between Glx and FWHM, as QM are colinear, and thus controlling for one in subsequent analysis is sufficient. Only significant correlations are reported. Figure S2: Quality of paired HERMES (GABA‐DIFF & SUM) and PRESS spectra acquired from the thalamus voxel significantly differs. Friedman one‐way repeated measure analysis was used to assess if there were significant differences in the quality of spectra (HERMES GABA‐DIFF (DIFF), HERMES SUM (SUM) and PRESS) acquired from the same voxel (in the same participant). Pairwise Wilcoxon rank sum tests were used for post hoc testing to isolate specific differences in quality metrics between paired spectra, with Bonferroni correction for multiple comparisons. Results of post hoc testing are shown for the thalamus voxel, with p values adjusted for multiple‐comparisons using Bonferroni correction. Ns: Nonsignificant, [file NBM-39-e70171-s001.docx]

### **Supplementary Table 1. Sequence acquisition parameters per scanner.**

| Scanner | Site | Scanner features | Structural (T1w) sequence | PRESS | HERMES |
| --- | --- | --- | --- | --- | --- |
| Scanner 1 | Nijmegen | Manufacturer: Siemens  Model: Skyra 3T  software: Syngo MR D13 | TR/TE/TI: 2300 / 3 / 900ms  Voxels size = 1.1 × 1.1 × 1.2 mm  Flip angle = 9°  Matrix size = 256 × 256  FOV = 270 mm  176 slices | TR/TE: 2000/35ms  Averages: 64, 16 interleaved water reference scans.  Water suppression:  Voxel dimensions:  Thalamus - 26 x 40 x 24 mm  ACC - 35 x 30 x 25 mm  Flip angle: 90 degrees  Datapoints: 4096 at 4000 Hz | TR/TE: 2000/80ms  Averages: 240 (60 averaged for A, B, C and D), 16 interleaved water reference scans.  Voxel dimensions:  Thalamus - 26 x 40 x 24 mm  ACC - 35 x 30 x 25 mm  Flip angle: 90 degrees  Editing pulses: 20 ms editing pulses placed at 1.90 ppm, 4.56 ppm and 7.46 ppm.  Datapoints: 4096 at 4000 Hz |
| Scanner 2 | Manheim | Manufacturer: Siemens  Model: TimTrio 3T  software: Syngo MR B17 | TR/TE/TI: 2300/2.95/900ms  Voxels size = 1.1 × 1.1 × 1.2 mm  Flip angle = 9°  Matrix size = 256 × 256  FOV = 270 mm  176 slices | TR/TE: 2000/35ms  Averages: 64, 16 interleaved water reference scans.  Voxel dimensions:  Thalamus - 30 x 30 x 30 mm  ACC - 35 x 30 x 25 mm  Flip angle: 90 degree   Datapoints: 4096 at 4000 Hz | TR/TE: 2000/80ms  Averages: 240 metabolite (60 averaged for A, B, C and D), 16 interleaved water reference scans.    Voxel dimensions:  Thalamus - 30 x 30 x 30 mm  ACC - 35 x 30 x 25 mm  Flip angle: 90 degree  Editing pulses: 20 ms editing pulses placed at 1.90 ppm, 4.56 ppm and 7.46 ppm.  Datapoints: 4096 at 4000 Hz |
| Scanner 3 | Nijmegen | Manufacturer: Siemens  Model: Magnetom Primsa 3T  software: prisma fit | TR/TE/TI: 2300 / 3 / 900ms  Voxels size = 1.1 × 1.1 × 1.0 mm  Flip angle = 9°  Matrix size = 256 × 256  FOV = 256 mm  208 slices | TR/TE: 2000/35ms  Averages: 64, 16 interleaved water reference scans.  Water suppression  Voxel dimensions:  Thalamus - 26 x 40 x 24 mm  Flip angle: 90 degrees  Datapoints: 4096 at 4000 Hz | TR/TE: 2000/80ms  Averages: 240 metabolite (60 averaged for A, B, C and D), 16 interleaved water reference scans.  Voxel dimensions:  Thalamus - 26 x 40 x 24 mm  Flip angle: 90 degrees  Editing pulses: 20 ms editing pulses placed at 1.90 ppm, 4.56 ppm and 7.46 ppm.  Datapoints: 4096 at 4000 Hz |

### **Supplementary Table 2. Data excluded per scanner and why.**

| Scanner | Data before QC  total (thalamus:ACC) | Data after QC  total (thalamus:ACC) | Data excluded and why  (thalamus:ACC) |
| --- | --- | --- | --- |
| Scanner 1 | 192 (96:96) | 164 (76:88) | 28 (20:8)  movement artefacts n = 27  lipid contamination n = 1 |
| Scanner 2 | 60 (31:29) | 56 (28:29) | 4 (4 thalamus only)  movement artefacts n = 4 |
| Scanner 3 | 16 (thalamus only) | 15 (thalamus only) | 1 (1 thalamus only)  movement artefacts n = 1 |
| Overall | 268 (143:125) | 235 (119:117) | 33 (25: 8)  movement artefacts n = 31  lipid contamination n = 1 |

## **Supplementary Table 3. Quality of thalamus and ACC PRESS and HERMES spectra per scanner.**

Differences in the quality of MRS data per scanner were assessed using Kruskal-Wallis tests, the calculated H statistics and corresponding p values are displayed in the table, with p_adjusted_ < 0.05 indicating a significant difference in the quality of MRS data per scanner after Bonferroni correction. Note SNR: signal to noise ratio of total creatine and FWHM: full width half maximum of total creatine.

|  | SNR -  HERMES | FWHM (Hz) – HERMES | Frequency shift (Hz)- HERMES | Fit residuals – HERMES GABA-DIFF | Fit residuals – HERMES SUM | SNR -  PRESS | FWHM (Hz) – PRESS | Frequency shift (Hz) - PRESS | Fit residuals – PRESS |
| --- | --- | --- | --- | --- | --- | --- | --- | --- | --- |
| Thalamus |  |  |  |  |  |  |  |  |  |
| Scanner 1 | 73.68 (26.24) | 9.08 (4.07) | -2.33 (3.06) | 2.46 (0.89) | 4.94 (2.29) | 67.63 (18.38) | 8.31 (1.75) | -4.02 (2.95) | 8.99 (11.57) |
| Scanner 2 | 62.04 (17.16) | 11.32 (3.33) | -2.44 (5.23) | 4.15 (3.42) | 10.71 (8.77) | 72.83 (14.42) | 8.82 (1.34) | -4.38 (1.86) | 4.82 (11.15) |
| Scanner 3 | 107.50 (56.76) | 6.78 (5.27) | -3.42 (1.44) | 1.99 (1.41) | 7.09 (2.30) | 87.79 (45.11) | 7.42 (5.37) | -2.76 (1.61) | 3.63 (3.052) |
| Kruskal-Wallis test for differences between scanners | H(3) = 31.15, p_adjusted_ = 0.00 | H(3) = 27.21, p_adjusted_ = 0.00 | H(3) = 8.14, p_adjusted_ = 0.22 | H(3) = 37.92, p_adjusted_ = 0.00 | H(3) = 43.99, p_adjusted_ = 0.00 | H(3) = 21.36, p_adjusted_ = 0.00 | H(3) = 16.48, p_adjusted_ = 0.005 | H(3) = 19.10, p_adjusted_ = 0.001 | H(3) = 7.44, p_adjusted_ = 0.22 |
| ACC |  |  |  |  |  |  |  |  |  |
| Scanner 1 | 229.53 (74.47) | 5.25 (1.66) | -2.62 (2.98) | 3.19 (1.09) | 16.66 (7.80) | 213.31 (53.99) | 5.20 (0.76) | -2.74 (1.67) | 9.10 (10.07) |
| Scanner 2 | 245.51 (49.94) | 5.22 (1.42) | -3.40 (1.95) | 3.19 (0.97) | 19.99 (8.76) | 251.36 (47.39) | 4.70 (0.29) | -4.21 (0.96) | 9.39 (2.082) |
| Kruskal-Wallis test for differences between scanners | H(2) = 16.43, p_adjusted_ = 0.001 | H(2) = 9.17, p_adjusted_ = 0.05 | H(2) = 6.79, p_adjusted_ = 0.16 | H(2) = 11.78, p_adjusted_ = 0.01 | 2) = 1.89, p_adjusted_ = 1 | H(2) = 43.39, p_adjusted_ = 0.00 | H(2) = 33.60, p_adjusted_ = 0.00 | H(2) = 21.84, p_adjusted_ = 0.00 | H(2) = 0.98, p_adjusted_ = 1 |


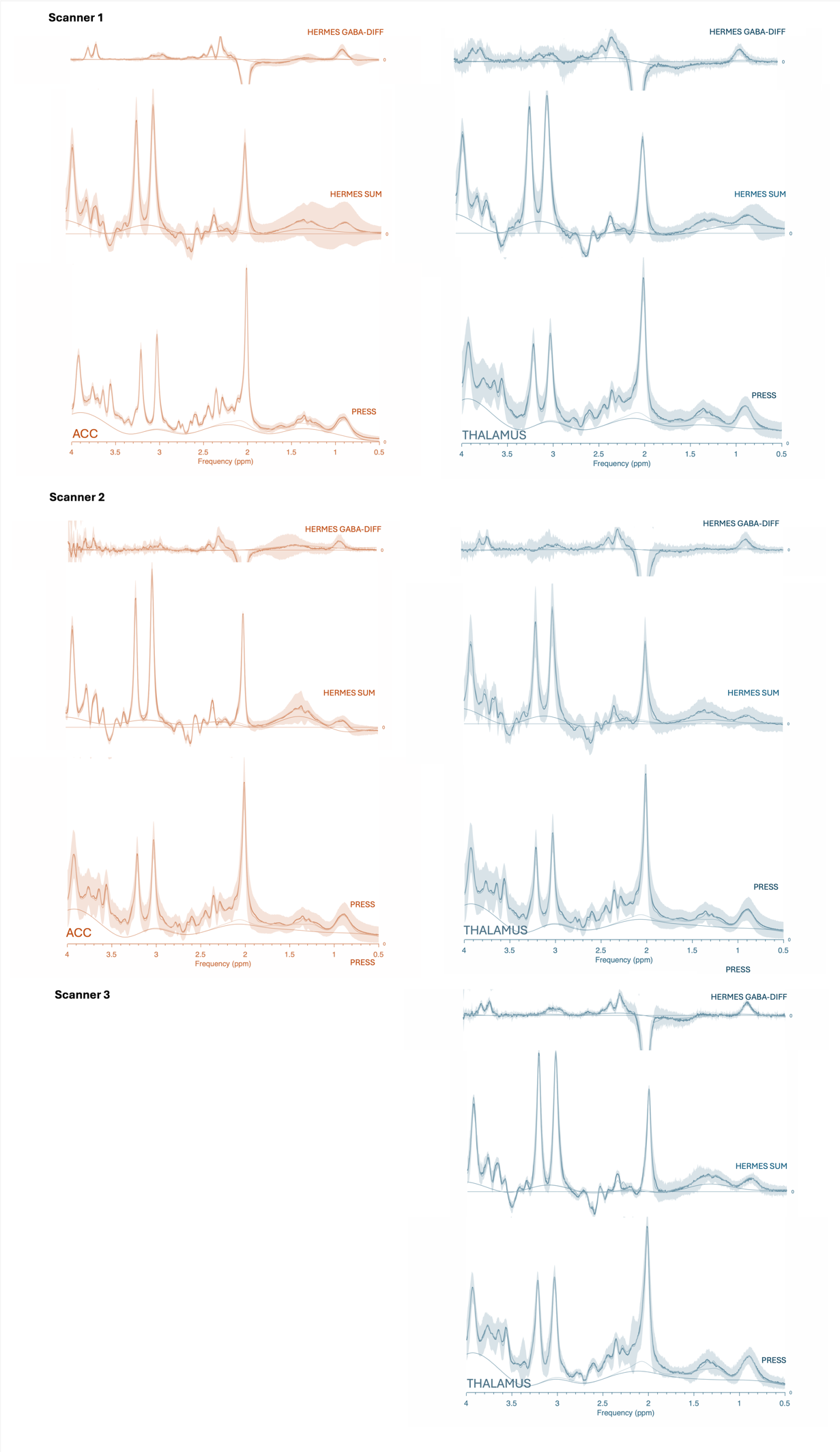


### **Supplementary Figure 1. Mean HERMES GABA-DIFF, HERMES SUM and PRESS spectra per scanner, showing model fit, model baseline and median residual fits (error bars).**

## **Supplementary Table 4. Significant correlations between FWHM and Glx estimates from HERMES SUM, HERMES GABA-DIFF and PRESS spectra.**

Correlations between FWHM and Glx estimates was assessed using Spearman’s Rank correlation coefficients. Correlation coefficients are displayed in the table, with p_adjusted_ < 0.05 indicating significance after Bonferroni correction. Note we chose to only observe correlations between Glx and FWHM, as QM are colinear, and thus controlling for one in subsequent analysis is sufficient. Only significant correlations are reported.

| Scanner & Voxel | x | y | cor | p.adj | method |
| --- | --- | --- | --- | --- | --- |
| Scanner 1 Thalamus | Glx_sum_creatinescaled | Cr_FWHM | -0.64 | 0 | Spearman |
| Scanner 1 Thalamus | Glx_sum_Tissurcorwaterscaled | Cr_FWHM | -0.7 | 0 | Spearman |
| Scanner 1 ACC | Glx_sum_creatinescaled | Cr_FWHM | -0.69 | 0 | Spearman |
| Scanner 1 ACC | Glx_sum_Tissurcorwaterscaled | Cr_FWHM | -0.8 | 0 | Spearman |
| Scanner 2 ACC | Glx_sum_Tissurcorwaterscaled | Cr_FWHM | -0.77 | 2.6E-05 | Spearman |
| Scanner 2 ACC | Glx_sum_creatinescaled | Cr_FWHM | -0.76 | 3.264E-05 | Spearman |
| Scanner 3 Thalamus | Glx_sum_Tissurcorwaterscaled | Cr_FWHM | -0.77 | 0.00864 | Spearman |

### **Supplementary Figure 2. Quality of paired HERMES (GABA-DIFF & SUM) and PRESS spectra acquired from the thalamus voxel significantly differs.**

Friedman One-Way Repeated Measure Analysis was used to assess if there were significant differences in the quality of spectra (HERMES GABA-DIFF (DIFF), HERMES SUM (SUM) and PRESS) acquired from the same voxel (in the same participant). Pairwise Wilcoxon Rank sum tests were used for post hoc testing to isolate specific differences in quality metrics between paired spectra, with Bonferroni correction for multiple comparisons. Results of post-hoc testing are shown for the thalamus voxel, with p-values adjusted for multiple-comparisons using Bonferroni correction. Ns: Nonsignificant, *P_adjusted_ < 0.05, ** P_adjusted_  < 0.01, *** P_adjusted_  < 0.001, **** P_adjusted_  < 0.0001, SNR: signal to noise ratio of total creatine, FWHM: full width half maximum of total creatine.

### **Supplementary Figure 3. Quality of paired HERMES (GABA-DIFF & SUM) and PRESS spectra acquired from the ACC voxel significantly differs.**

Friedman One-Way Repeated Measure Analysis was used to assess if there were significant differences in the quality of spectra (HERMES GABA-DIFF (DIFF), HERMES SUM (SUM) and PRESS) acquired from the same voxel (in the same participant). Pairwise Wilcoxon Rank sum tests were used for post hoc testing to isolate specific differences in quality metrics between paired spectra, with Bonferroni correction for multiple comparisons. Results of post-hoc testing are shown for the ACC voxel, with p-values adjusted for multiple-comparisons using Bonferroni correction. Ns: Nonsignificant, *P_adjusted_ < 0.05, ** P_adjusted_  < 0.01, *** P_adjusted_  < 0.001, **** P_adjusted_  < 0.0001, SNR: signal to noise ratio of total creatine, FWHM: full width half maximum of total creatine.

### **Supplementary Figure 4. The quality of MRS data (HERMES (GABA-DIFF & SUM) and PRESS) acquired from the same participant differs between thalamus and ACC voxels.**

Friedman One-Way Repeated Measure Analysis were used to assess if quality of MRS data (HERMES (DIFF & SUM) and PRESS) acquired from the same participant significantly differed between voxels (thalamus and ACC). Pairwise Wilcoxon Rank sum tests were used for post hoc testing where appropriate, with Bonferroni correction for multiple comparisons. Results of post-hoc testing are shown, with p-values adjusted for multiple-comparisons using Bonferroni correction. Ns: Nonsignificant, *P_adjusted_ < 0.05, ** P_adjusted_  < 0.01, *** P_adjusted_  < 0.001, **** P_adjusted_  < 0.0001. Note scanner 4 did not record data from an ACC voxel.

### **Supplementary Figure 5. Glx (creatine-scaled (/tCr) and tissue-corrected (i.u)) concentrations quantified from the PRESS, HERMES GABA-DIFF (DIFF) and HERMES SUM spectra per scanner for the ACC and thalamus voxels.**

Kruskal-Wallis tests were used to assess if PRESS, HERMES DIFF and HERMES SUM estimated Glx concentrations differed between scanners 1 – 3 for the thalamus and ACC voxels. Mann-Whitney-Wilcoxon tests were used for post hoc testing to isolate specific differences between scanners where appropriate, with Bonferroni correction for multiple comparisons. Results of post-hoc testing are shown. Ns: Nonsignificant, *P < 0.05, **P < 0.01, ***P < 0.001, ****P < 0.0001. Note scanner 3 did not record data from an ACC voxel.

### **Supplementary Table 5. ICC values (and 95% confidence intervals) calculated between HERMES (GABA-DIFF and SUM) and PRESS Glx estimates per voxel per scanner for tissue-corrected and creatine-scaled data.**

Note overall refers to data pooled across voxels.

| Site Glx | PRESS:HERMES GABA-DIFF | PRESS:HERMES SUM | HERMES DIFF:HERMES SUM |
| --- | --- | --- | --- |
| **Creatine-scaled** |  |  |  |
| Scanner 1 | Thalamus = 0.047 (-0.179 - 0.27) poor  ACC = 0.23 (0.024 - 0.42) poor  Overall = 0.120 poor | Thalamus = 0.106 (-0.121 - 0.323) poor  ACC = 0.149 (-0.061 - 0.35) poor  Overall = 0.53 moderate | Thalamus = 6.2e-16 (-0.224 - 0.224) poor  ACC = 0.00 (-0.208 - 0.208) poor  Overall = 0.0204 poor |
| Scanner 2 | Thalamus = 0.028 (-0.3 -0.398) poor  ACC = 0.110 (-0.261 - 0.45) poor  Overall = 0.00 poor | Thalamus = 0.092 (-0.29 - 0.45) poor  ACC = 0.247 (-0.126 - 0.56) poor  Overall = 0.337 poor | Thalamus = 0.59 (0.276 - 0.79) moderate  ACC = 0.00 (-0.361 - 0.361) poor  Overall = 0.36 poor |
| Scanner 3 | Thalamus = 2.2e-10 (-0.50 - 0.497) poor | Thalamus = 0.86 (0.624 - 0.95) moderate | Thalamus = 0.0 ( -0.497 - 0.50) poor |
| **Tissue-corrected** |  |  |  |
| Scanner 1 | Thalamus = 0.00 (-0.224 - 0.22) poor  ACC = 0.059 (-0.15 - 0.26) poor  Overall = 0.053 poor | Thalamus = 0.260 (0.038 - 0.46) poor  ACC = 0.312 (0.110 - 0.488) poor  Overall = 0.71 moderate | Thalamus = 0.00 (-0.224 - 0.224) poor  ACC = 0.0319 (-0.178 - 0.239) poor  Overall = 0.00 poor |
| Scanner 2 | Thalamus = 0.175 (-0.21 - 0.52) poor  ACC = 0.44 (0.094 - 0.69) poor  Overall = 0.074 poor | Thalamus = 0.105 (-0.280 - 0.461) poor  ACC = 0.574 (0.269 - 0.77) moderate  Overall = 0.54 moderate | Thalamus = (-0.0040 - 0.65 ) 0.37 poor  ACC = 0.72 (0.488 - 0.859) moderate  Overall = 0.363 poor |
| Scanner 3 | Thalamus = 0.00 (-0.50 - 0.50) poor | Thalamus = 0.66 (0.236 - 0.87) moderate | Thalamus = 0.00 (-0.497 - 0.50) poor |

## **Supplementary Table 6. Quality of thalamus and ACC PRESS and HERMES spectra per scanner per diagnosis.**

Differences in the quality of MRS data per scanner were assessed using Kruskal-Wallis tests, the calculated H statistics and corresponding p values are displayed in the table, with p < 0.05 indicating a significant difference in the quality of MRS data per scanner. Note SNR: signal to noise ratio of total creatine and FWHM: full width half maximum of total creatine.

|  | SNR -  HERMES | FWHM – HERMES | Frequency shift- HERMES | Fit residuals – HERMES GABA-DIFF | Fit residuals – HERMES SUM | SNR -  PRESS | FWHM – PRESS | Frequency shift - PRESS | Fit residuals – PRESS |
| --- | --- | --- | --- | --- | --- | --- | --- | --- | --- |
| Thalamus |  |  |  |  |  |  |  |  |  |
| Scanner 1 TD | 71.08 (28.85) | 9.98 (4.77) | -1.39 (4.96) | 2.62 (0.88) | 4.43 (2.11) | 63.97 (19.34) | 8.39 (1.39) | -3.49 (2.91) | 8.32 (13.88) |
| Scanner 2 TD | 59.64 (7.29) | 11.28 (3.02) | -2.53  (3.29) | 5.08 (6.08) | 10.83 (7.12) | 70.21 (15.11) | 8.73 (1.61) | \| -4.34 \| \| --- \|   (1.03) | 13.67 (28.4) |
| Scanner 1 autism | 75.50 (25.44) | 8.88 (3.66) | -2.56 (3.49) | 2.37 (0.93) | 5.14 (2.35) | 69.51 (15.85) | 8.30 (2.48) | -4.32 (2.14) | 9.31 (10.41) |
| Scanner 2 autism | 69.34 (21.06) | 11.96 (3.19) | -1.53 (6.61) | 4.21 (2.63) | 9.21 (5.76) | 79.28 (19.01) | 8.64 (1.14) | -5.16 (1.97) | 4.25 (2.01) |
| ACC |  |  |  |  |  |  |  |  |  |
| Scanner 1 TD | 229.33 (58.07) | 5.17 (1.06) | -1.76 (4.92) | 3.11 (1.15) | 17.47 (7.39) | 207.79 (48.55) | 5.016 (0.75) | -2.43 (1.87) | 9.11 (10.26) |
| Scanner 2 TD | 252.15 (48.97) | 4.94 (1.16) | -3.65 (0.99) | 2.98 (0.99) | 18.24 (9.00) | 250.62 (44.54) | 4.59 (0.39) | -4.29 (0.96) | 9.35 (2.50) |
| Scanner 1 autism | 230.75 (79.10) | 5.356 (1.80) | -2.732 (1.88) | 3.22 (1.01) | 16.39 (8.25) | 213.91 (56.94) | 5.21 (0.73) | -3.28 (1.89) | 8.54 (10.67) |
| Scanner 2 autism | 239.63 (59.49) | 5.81 (1.147) | -2.912 (1.64) | 3.28 (0.66) | 20.81 (8.23) | 247.49 (61.40) | 4.80 (0.70) | -4.19 (1.01) | 9.29 (1.77) |

## **Supplementary Table 7. Glx concentrations quantified from the thalamus and ACC PRESS and HERMES spectra per scanner per diagnosis.**

|  | Glx/tCr PRESS | Glx/tCr HERMES GABA-DIFF | Glx/tCr  HERMES SUM | Glx (i.u)  PRESS | Glx (i.u)  HERMES DIFF | Glx (i.u)  HERMES SUM |
| --- | --- | --- | --- | --- | --- | --- |
| Thalamus |  |  |  |  |  |  |
| Scanner 1 TD | 1.15 (0.19) | 1.90 (0.46) | 0.45 (0.31) | 13.11 (1.91) | 19.81 (5.05) | 4.49 (2.65) |
| Scanner 2 TD | 0.90 (0.18) | 1.99 (0.74) | 0.48 (0.23) | 11.36 (4.53) | 18.18 (7.07) | 4.21 (1.45) |
| Scanner 1 autism | 1.08 (0.23) | 1.68 (0.51) | 0.47 (0.31) | 13.68 (3.50) | 17.77 (4.48) | 4.80 (2.98) |
| Scanner 2 autism | 0.93 (0.10) | 2.17 (1.66) | 0.50 (0.22) | 11.65 (2.35) | 20.29 (7.24) | 4.64 (1.97) |
| ACC |  |  |  |  |  |  |
| Scanner 1 TD | 1.39 (0.11) | 1.85 (0.29) | 0.98 (0.20) | 19.16 (2.19) | 19.17 (2.35) | 9.71 (2.18) |
| Scanner 2 TD | 1.42 (0.15) | 1.67 (0.13) | 0.99 (0.31) | 20.97 (1.96) | 16.56(1.82) | 9.49 (3.05) |
| Scanner 1 autism | 1.39 (0.15) | 1.82 (0.22) | 0.90 (0.24) | 19.31 (2.12) | 18.70 (3.42) | 9.13 (2.98) |
| Scanner 2 autism | 1.40 (0.10) | 1.72 (0.087) | 0.84 (0.35) | 18.99 (3.11) | 17.26 (4.60) | 7.9 (3.69) |

## **Supplementary Figure 6. Thalamus and ACC Glx concentrations (creatine-scaled (/tCr) and tissue-corrected (i.u)) estimated from the HERMES DIFF, HERMES SUM and PRESS spectra per scanner per diagnostic group.**

Tissue corrected (i.u) and creatine-scaled (/tCr) Glx concentrations estimated from the HERMES DIFF (DIFF), HERMES SUM (SUM) and PRESS spectra are shown per voxel, per scanner and per diagnostic group (TD and autism). Friedman One-Way Repeated Measure Analysis was used to assess if there were significant differences in the concentration of Glx estimated from the HERMES DIFF (DIFF), HERMES SUM (SUM) and PRESS spectra acquired from the same voxel per diagnostic group. Pairwise Wilcoxon Rank sum tests were used for post hoc testing where appropriate to isolate specific differences in Glx concentration between paired spectra, with Bonferroni correction for multiple comparisons. Results of post-hoc testing are shown per voxel.

## **Supplementary Figure 7. Bland-Altman (Giavarina version) plots comparing Glx concentrations estimated from paired HERMES GABA-DIFF (Glx DIFF) and PRESS spectra acquired from a thalamus voxel on scanner 1 for autism and TD groups**.

Bland-Altman (Giavarina version) plots comparing creatine-scaled (/tCr) and tissue-corrected (i.u) Glx estimated from paired HERMES DIFF and PRESS spectra from the thalamus voxel, scanner 1, for autism and TD groups. For each plot, the percentage difference between paired measures is shown on the y-axis, while the mean of the paired measures is shown the x axis. Dashed lines represent the overall mean percentage difference (estimated bias), the upper and lower limits of agreement (overall mean difference ± 1.96 standard deviation). Confidence intervals for limits of agreement are also shown. Linear regressions (model = percentage difference of paired measured ~ mean of paired measures) were used to identify proportional bias between paired measures, the resulting beta coefficient and corresponding p value is shown in the right-hand corner of each plot.

## **Supplementary Figure 8. Bland-Altman (Giavarina version) plots comparing Glx concentrations estimated from paired HERMES GABA-DIFF and PRESS spectra acquired from a ACC voxel on scanner 1 for autism and TD groups.**

Bland-Altman (Giavarina version) plots comparing creatine-scaled (/tCr) and tissue-corrected (i.u) Glx estimated from paired HERMES DIFF and PRESS spectra from the ACC voxel, scanner 1, for autism and TD groups. For each plot, the percentage difference between paired measures is shown on the y-axis, while the mean of the paired measures is shown the x axis. Dashed lines represent the overall mean percentage difference (estimated bias), the upper and lower limits of agreement (overall mean difference ± 1.96 standard deviation). Confidence intervals for limits of agreement are also shown. Linear regressions (model = percentage difference of paired measured ~ mean of paired measures) were used to identify proportional bias between paired measures, the resulting beta coefficient and corresponding p value is shown in the right-hand corner of each plot.

## **Supplementary Figure 9. Bland-Altman (Giavarina version) plots comparing Glx concentrations estimated from paired HERMES DIFF and PRESS spectra acquired from a thalamus voxel on scanner 2 for autism and TD groups.**

Bland-Altman (Giavarina version) plots comparing creatine-scaled (/tCr) and tissue-corrected (i.u) Glx estimated from paired HERMES DIFF and PRESS spectra from the thalamus voxel, scanner 2, for autism and TD groups. For each plot, the percentage difference between paired measures is shown on the y-axis, while the mean of the paired measures is shown the x axis. Dashed lines represent the overall mean percentage difference (estimated bias), the upper and lower limits of agreement (overall mean difference ± 1.96 standard deviation). Confidence intervals for limits of agreement are also shown. Linear regressions (model = percentage difference of paired measured ~ mean of paired measures) were used to identify proportional bias between paired measures, the resulting beta coefficient and corresponding p value is shown in the right-hand corner of each plot.

## **Supplementary Figure 10. Bland-Altman (Giavarina version) plots comparing Glx concentrations estimated from paired HERMES GABA-DIFF and PRESS spectra acquired from an ACC voxel on scanner 2 for autism and TD groups.**

Bland-Altman (Giavarina version) plots comparing creatine-scaled (/tCr) and tissue-corrected (i.u) Glx estimated from paired HERMES DIFF and PRESS spectra from the ACC voxel, scanner 2, for autism and TD groups. For each plot, the percentage difference between paired measures is shown on the y-axis, while the mean of the paired measures is shown the x axis. Dashed lines represent the overall mean percentage difference (estimated bias), the upper and lower limits of agreement (overall mean difference ± 1.96 standard deviation). Confidence intervals for limits of agreement are also shown. Linear regressions (model = percentage difference of paired measured ~ mean of paired measures) were used to identify proportional bias between paired measures, the resulting beta coefficient and corresponding p value is shown in the right-hand corner of each plot.

## **Supplementary Figure 11. Bland-Altman (Giavarina version) plots comparing Glx concentrations estimated from paired HERMES SUM and PRESS spectra acquired from a thalamus voxel on scanner 1 for autism and TD groups.**

Bland-Altman (Giavarina version) plots comparing creatine-scaled (/tCr) and tissue-corrected (i.u) Glx estimated from paired HERMES SUM and PRESS spectra from the thalamus voxel, scanner 1, for autism and TD groups. For each plot, the percentage difference between paired measures is shown on the y-axis, while the mean of the paired measures is shown the x axis. Dashed lines represent the overall mean percentage difference (estimated bias), the upper and lower limits of agreement (overall mean difference ± 1.96 standard deviation). Confidence intervals for limits of agreement are also shown. Linear regressions (model = percentage difference of paired measured ~ mean of paired measures) were used to identify proportional bias between paired measures, the resulting beta coefficient and corresponding p value is shown in the right-hand corner of each plot.

## **Supplementary Figure 12. Bland-Altman (Giavarina version) plots comparing Glx concentrations estimated from paired HERMES SUM and PRESS spectra acquired from an ACC voxel on scanner 1 for autism and TD groups.**

Bland-Altman (Giavarina version) plots comparing creatine-scaled (/tCr) and tissue-corrected (i.u) Glx estimated from paired HERMES SUM and PRESS spectra from the ACC voxel, scanner 1, for autism and TD groups. For each plot, the percentage difference between paired measures is shown on the y-axis, while the mean of the paired measures is shown the x axis. Dashed lines represent the overall mean percentage difference (estimated bias), the upper and lower limits of agreement (overall mean difference ± 1.96 standard deviation). Confidence intervals for limits of agreement are also shown. Linear regressions (model = percentage difference of paired measured ~ mean of paired measures) were used to identify proportional bias between paired measures, the resulting beta coefficient and corresponding p value is shown in the right-hand corner of each plot.

## **Supplementary Figure 13. Bland-Altman (Giavarina version) plots comparing Glx concentrations estimated from paired HERMES SUM and PRESS spectra acquired from a thalamus voxel on scanner 2 for autism and TD groups. Chnag 2**

Bland-Altman (Giavarina version) plots comparing creatine-scaled (/tCr) and tissue-corrected (i.u) Glx estimated from paired HERMES SUM and PRESS spectra from the thalamus voxel, scanner 2, for autism and TD groups. For each plot, the percentage difference between paired measures is shown on the y-axis, while the mean of the paired measures is shown the x axis. Dashed lines represent the overall mean percentage difference (estimated bias), the upper and lower limits of agreement (overall mean difference ± 1.96 standard deviation). Confidence intervals for limits of agreement are also shown. Linear regressions (model = percentage difference of paired measured ~ mean of paired measures) were used to identify proportional bias between paired measures, the resulting beta coefficient and corresponding p value is shown in the right-hand corner of each plot.

## **Supplementary Figure 14. Bland-Altman (Giavarina version) plots comparing Glx concentrations estimated from paired HERMES SUM and PRESS spectra acquired from an ACC voxel on scanner 2 for autism and TD groups.**

Bland-Altman (Giavarina version) plots comparing creatine-scaled (/tCr) and tissue-corrected (i.u) Glx estimated from paired HERMES SUM and PRESS spectra from the ACC voxel, scanner 2, for autism and TD groups. For each plot, the percentage difference between paired measures is shown on the y-axis, while the mean of the paired measures is shown the x axis. Dashed lines represent the overall mean percentage difference (estimated bias), the upper and lower limits of agreement (overall mean difference ± 1.96 standard deviation). Confidence intervals for limits of agreement are also shown. Linear regressions (model = percentage difference of paired measured ~ mean of paired measures) were used to identify proportional bias between paired measures, the resulting beta coefficient and corresponding p value is shown in the right-hand corner of each plot.

## **Supplementary Table 8. Bland-Altman regression analysis by diagnosis.**

The table shows the results from linear regressions (model = percentage difference of paired measured ~ mean of paired measures) used to identify proportional bias between paired measures. Significant beta coefficients indicate significant proportional bias (as the magnitude of Glx changes, the disparity between measurements changes). NS = non-significant proportional bias.

| Linear regression | PRESS: HERMES GABA-DIFF | PRESS: HERMES SUM |
| --- | --- | --- |
| **Creatine-scaled** |  |  |
| Scanner 1 Autism | Thalamus: beta = 1.50, p < 0.05  ACC: beta = 1.33, p < 0.05 | Thalamus: beta _=_ 0.90, p < 0.05  ACC: beta _=_ 0.81, p < 0.05 |
| Scanner 1 TD | Thalamus: beta _=_ 1.13; p < 0.05  ACC: beta = 1.13, p < 0.05 | Thalamus: NS  ACC: beta = 0.90, p < 0.05 |
| Scanner 2 Autism | Thalamus: beta = 1.89, p < 0.05  ACC: beta = 1.20, p < 0.05 | Thalamus: beta = 1.44, p < 0.05  ACC: beta = 1.31, p < 0. |
| Scanner 2 TD | Thalamus: beta = 1.20, p < 0.05  ACC: NS | ACC: beta = 0.904, p < 0.05  Thalamus: NS |
| **Tissue-corrected** |  |  |
| Scanner 1 Autism | Thalamus: NS  ACC: NS | Thalamus: NS  ACC: NS |
| Scanner 1 TD | Thalamus: NS  ACC: NS | Thalamus: NS  ACC: NS |
| Scanner 2 Autism | Thalamus: NS  ACC: NS | Thalamus: NS  ACC: beta = -1.31, p < 0.05 |
| Scanner 2 TD | Thalamus: NS  ACC: beta = -0.79, p < 0.05 | Thalamus: NS  ACC: NS |

## **Supplementary Table 9. ICC values calculated between HERMES (DIFF and SUM) and PRESS Glx estimates per voxel per scanner for tissue-corrected and creatine-scaled data per diagnostic group.**

| Scanner | PRESS: HERMES GABA-DIFF | PRESS: HERMES SUM |
| --- | --- | --- |
| **Creatine-scaled** |  |  |
| Scanner 1 Autism | Thalamus = 0.0 (-0.29 - 0.29) poor  ACC = 0.25 (-0.014 - 0.49) poor | Thalamus = 0.029 (-0.26 - 0.31) poor  ACC = 0.24 (-0.031 - 0.48) poor |
| Scanner 1 TD | Thalamus = 0.12 (-0.23 - 0.45) poor  ACC = 0.11 (-0.21 - 0.42) poor | Thalamus = 0.234 (-0.12 - 0.54) poor  ACC = 0.00 (-0.32 - 0.32) poor |
| Scanner 2 Autism | Thalamus = 0.0 (-0.51 - 0.51) poor  ACC = 00.14 (-0.39 - 0.61) poor | Thalamus = 0.00 (-0.51 - 0.51) poor  ACC = 0.14 (-0.40 - 0.61) poor |
| Scanner 2 TD | Thalamus = 0.47 (-0.18 - 0.83) poor  ACC = 0.12 (-0.44 - 0.62) poor | Thalamus = 0.64 (0.069 - 0.90) moderate  ACC = 0.37 (-0.19 - 0.76) poor |
| **Tissue-corrected** |  |  |
| Scanner 1 Autism | Thalamus = 0.00 (-0.29 - 0.29) poor  ACC = 0.034 (-0.24 - 0.30) poor | Thalamus = 0.24 (-0.050 - 0.50) poor  ACC = 0.43 (0.18 - 0.63) poor |
| Scanner 1 TD | Thalamus =0.030 (-0.32 - 0.38) poor  ACC = 0.17 (-0.16 - 0.47) poor | Thalamus = 0.29 (-0.060 - 0.58) poor  ACC =0.039 (-0.28 - 0.35) poor |
| Scanner 2 Autism | Thalamus = 0.099 (-0.44 - 0.58) poor  ACC = 0.57 (0.073 - 0.84) moderate | Thalamus = 0.00 (-0.51 - 0.51) poor  ACC = 0.63 (0.16 - 0.86) moderate |
| Scanner 2 TD | Thalamus = 0.43 (-0.23 - 0.82) poor  ACC =0.49 (-0.048 - 0.81) poor | Thalamus = 0.65 (0.09 - 0.90) moderate  ACC = 0.55 (0.032 - 0.84) moderate |

## **Supplementary Table 10. Inter-subject CoV of Glx estimates from HERMES (DIFF and SUM) and PRESS Glx spectra per scanner for tissue-corrected and creatine-scaled data from the thalamus and ACC, per diagnostic group.**

| Glx | ACC (inter-subject CoV) | Thalamus (inter-subject CoV) | ACC (intre-subject CoV) | Thalamus (intre-subject CoV) |
| --- | --- | --- | --- | --- |
| Scanner 1 autism | Creatine scaled:  PRESS = 7.96  DIFF = 19.61  **SUM = 21.38**  Tissue corrected:  PRESS = 9.02 DIFF = 17.10  **SUM = 22.38** | Creatine scaled:  PRESS = 16.06  DIFF = 25.79  **SUM = 43.00**  Tissue corrected:  PRESS = 19.28 DIFF = 23.18  **SUM = 39.9** | Creatine scaled:  35.98  Tissue corrected:  **38.35** | Creatine scaled:  **57.00**  Tissue corrected:  55.85 |
| Scanner 1 - TD | Creatine scaled:  PRESS = 7.04  DIFF = 10.88  **SUM = 16.20**  Tissue corrected:  PRESS = 7.35  DIFF = 9.18  **SUM = 16.48** | Creatine scaled:  PRESS = 14.57  DIFF = 29.31  **SUM = 43.19**  Tissue corrected:  PRESS = 14.6 DIFF = 26.45  **SUM = 40.51** | Creatine scaled:  33.06  Tissue corrected:  **34.06** | Creatine scaled:  **62.65**  Tissue corrected:  60.22 |
| Scanner 2 - autism | Creatine scaled:  PRESS = 6.16  DIFF = 11.51  **SUM = 29.79**  Tissue corrected:  PRESS = 14.96  DIFF = 24.21  **SUM = 49.48** | Creatine scaled:  PRESS = 23.08  DIFF = 47.21  **SUM =82.64**  Tissue corrected:  PRESS = 45.53 DIFF = 41.25  **SUM = 54.17** | Creatine scaled:  35.41  Tissue corrected:  **40.045** | Creatine scaled:  **72.41**  Tissue corrected:  69.67 |
| Scanner 2 - TD | Creatine scaled:  PRESS = 6.39  DIFF = 9.20  **SUM = 20.40**  Tissue corrected:  PRESS = 19.25 DIFF =12.56  **SUM = 28.13** | Creatine scaled:  PRESS = 28.99  DIFF = 29.10  **SUM =52.06**  Tissue corrected:  PRESS = 29.80 DIFF = 32.28  **SUM = 53.12** | Creatine scaled:  27.70  Tissue corrected:  **39.18** | Creatine scaled:  **64.97**  Tissue corrected:  60.48 |
